# Parvalbumin Role in Epilepsy and Psychiatric Comorbidities: From Mechanism to Intervention

**DOI:** 10.3389/fnint.2022.765324

**Published:** 2022-02-17

**Authors:** Lívea Dornela Godoy, Tamiris Prizon, Matheus Teixeira Rossignoli, João Pereira Leite, José Luiz Liberato

**Affiliations:** ^1^Department of Psychology, Faculty of Philosophy, Sciences and Letters of Ribeirão Preto, University of São Paulo, Ribeirão Preto, Brazil; ^2^Department of Neuroscience and Behavioral Sciences, Ribeirão Preto Medical School, University of São Paulo, Ribeirão Preto, Brazil

**Keywords:** parvalbumin, epilepsy, psychiatric comorbidities, pharmacological interventions, non-pharmacological interventions, designer receptor exclusively activated by designer drugs (DREADD), optogenetic, cell therapy

## Abstract

Parvalbumin is a calcium-binding protein present in inhibitory interneurons that play an essential role in regulating many physiological processes, such as intracellular signaling and synaptic transmission. Changes in parvalbumin expression are deeply related to epilepsy, which is considered one of the most disabling neuropathologies. *Epilepsy* is a complex multi-factor group of disorders characterized by periods of hypersynchronous activity and hyperexcitability within brain networks. In this scenario, inhibitory neurotransmission dysfunction in modulating excitatory transmission related to the loss of subsets of parvalbumin-expressing inhibitory interneuron may have a prominent role in disrupted excitability. Some studies also reported that parvalbumin-positive interneurons altered function might contribute to psychiatric comorbidities associated with epilepsy, such as depression, anxiety, and psychosis. Understanding the epileptogenic process and comorbidities associated with epilepsy have significantly advanced through preclinical and clinical investigation. In this review, evidence from parvalbumin altered function in epilepsy and associated psychiatric comorbidities were explored with a translational perspective. Some advances in potential therapeutic interventions are highlighted, from current antiepileptic and neuroprotective drugs to cutting edge modulation of parvalbumin subpopulations using optogenetics, designer receptors exclusively activated by designer drugs (DREADD) techniques, transcranial magnetic stimulation, genome engineering, and cell grafting. Creating new perspectives on mechanisms and therapeutic strategies is valuable for understanding the pathophysiology of epilepsy and its psychiatric comorbidities and improving efficiency in clinical intervention.

## Introduction

Epilepsy is one of the most disabling chronic neurologic disorders, significantly impacting patients’ quality of life (Devinsky et al., 2018). Worldwide, around 70 million people have epilepsy (Singh and Trevick, 2016). In those patients initiating anti-seizure treatment, only around 60% will be seizure-free, and only a few more will achieve seizure control with polytherapy. Seizures that did not successfully control within the first pharmacological intervention may have greater odds of not responding to a subsequent medication regimen (Chen et al., 2018; Löscher et al., 2020).

Drug resistance in epilepsy also is associated with loss of productivity, employment, and significant direct and indirect health costs (Löscher et al., 2020). It could be further deleterious by its association with psychiatric comorbidities, which pose a relevant problem considering the high incidence and the increased pharmacoresistance in those patients (Hermann et al., 2000; Johnson et al., 2004; Kanner et al., 2018).

Epilepsy is a complex multifactorial group of disorders characterized by periods of hypersynchronous activity and hyperexcitability within brain networks. It is complex because it is not a single disease but the result of a wide range of underlying etiologies and pathologies (Sirven, 2015), all sharing the common and fundamental characteristic of predisposing the brain to manifest a pathologic and enduring tendency to generate epileptic seizures (Fisher et al., 2014).

The transient occurrence of signs and/or symptoms due to an abnormal excessive or synchronous neuronal activity in the brain defines an epileptic seizure (Beghi et al., 2005). Dysfunction in inhibitory neurotransmission and/or modulating excitatory transmission is related to the increased excitability in epilepsies (Cossart et al., 2005; Ben-Ari, 2006; Pitkänen and Engel, 2014). But the E/I imbalance is only the tip of the iceberg in epilepsy. This process includes cellular diversity, synaptic spatiotemporal dynamics of interneuronal connectivity, and circuit reorganization. Therefore, there are many epileptogenic plasticities related to epilepsies, not to mention, variability associated with psychiatric comorbidities (Marafiga et al., 2021).

Interneurons represent a crucial evolutionary step that enables different forms of computational processing and rapid dynamics. Among them, parvalbumin-positive interneurons (PV+) are one of the master regulators of excitation-inhibition balance and timing of principal cells, supporting different oscillation patterns (Buzsáki and Chrobak, 1995; Roux and Buzsáki, 2015; Fishell and Kepecs, 2020). Not only all of those special features contribute to determinant brain and behavior regular function but are also deeply altered in epilepsies (Jiang et al., 2016). Additionally, PV+ seems to be profoundly related to depression (Csabai et al., 2017) as well as anxiety and schizophrenia.

Therefore, PV+ interneurons may exert a convergent role, configuring an important link in epilepsy and psychiatric comorbidities. Promising discoveries could lead to a better understanding of the pathology mechanisms and determinant advances in the treatment. Therefore, advances in potential therapeutic interventions will be discussed, as we will present key findings on the parvalbumin role on epilepsy and epilepsy comorbidities.

## Methods

Considering the sui generis characteristics and its involvement in epilepsy and neuropsychiatric disorders, we integrated basic and clinical findings in a translational perspective, highlighting potential therapeutic strategies in this comprehensive revision. Using PubMed, we combined the descriptors *epilepsy(ies)* or *seizure(s)* with *parvalbumin*, *parvalbuminergic*, or *parvalbumin* with terms related to the respective sections. The detailed information can be found in the Supplementary File. With the exception of seminal communications, the great majority of intervention papers here cited are from the 1980’s to 2021.

## Parvalbumin: Structure and Function

**FIGURE 1:**
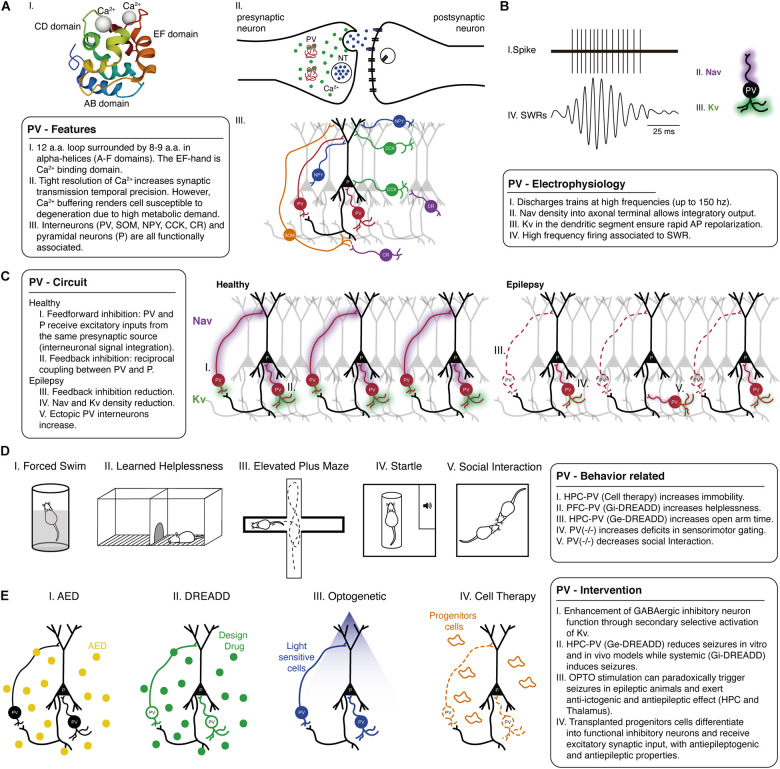
Parvalbumin role in epilepsy and psychiatric comorbidities: evidence from animal models. **(A)** Parvalbumin-positive interneurons show several special physiological features including, **(I)** PV is a Ca^2+^ binding protein with a specific structure and protein dynamics. **(II)** Tight Ca^2+^ homeostasis through parvalbumin binding enables regulated transmitter release and synaptic transmission temporal precision. **(III)** Such characteristics are also related to the configuration circuitry, including distinct inhibitory/excitatory cell types. Also, **(B)** Parvalbumin electrophysiological properties involving distribution and kinetics of Na^+^ voltage-dependent channel (purple shade) and K^+^ voltage-dependent channel (green shade) are related to fast-action characteristics that are critically important in brain rhythms related to several behavioral functions. **(C)** Parvalbumin circuitry can be composed of different forms of associations, and it is of particular importance **(I)** feedforward and **(II)** feedback pathways, as interneurons receive/send input from/to different connections. The mechanisms of parvalbumin-positive interneuron in healthy and **(III)** pathological conditions should be considered to tailor specific interventions to restore that dysfunctional host circuitry in epilepsy, such as restoring lost cells and connections (red dashed lines) and changes in electrophysiological properties (reduced purple and green shades). **(D)** In animal models, manipulating parvalbumin-positive interneurons also allows us to understand behavior related to psychiatric comorbidities. Epilepsy-associated behavioral deficits might be mitigated at earlier interventions and usually involve parvalbumin-positive interneurons in the prefrontal cortex, hippocampus, and amygdala. **(I)** Parvalbumin-positive interneuron precursors grafting into the hippocampus decreased immobility in the forced swim test. **(II)** Chemogenetic excitation of parvalbumin-positive interneurons in the prefrontal cortex can improve depressive-like behavior in learned helplessness, and **(III)** similarly, DREADD and cell therapy in hippocampus and amygdala improved anxiety-like behavior in epilepsy models by increasing time spent in the open arms of the elevated plus-maze. There are several abnormalities in the hippocampus and prefrontal cortex related to psychotic-like impairments and epilepsy. The generalized hypofunction in parvalbumin knockout mice can induce **(IV)** sensorimotor gating deficits in prepulse inhibition (PPI) and decrease social interaction time. **(E)** Parvalbumin-target interventions have been explored in neuroscience though **(I)** classical pharmacotherapy could be better designed to target this cell population or cutting-edge techniques such as **(II)** DREADD (green dots), **(III)** OPTO (blue light), and **(IV)** cell grafting therapy (orange progenitor cells). All those recent advances have shown that manipulating parvalbumin-positive interneuron function could promote a significant advance, not only in epilepsy therapy but also in psychiatric comorbidities. a.a., amino acids; AED, antiepileptic drug; AMY, amygdala; AP, action potential; Ca^2+^, calcium; CCK, cholecystokinin-positive cells; CR, calretinin-positive cells; DREADD, designer receptor exclusively activated by the designer drug; Ge-DREADD, DREADD excitation; Gi-DREADD, DREADD inhibition; HPC, hippocampus; K_*v*_, Potassium voltage-dependent channel; Na_*v*_, Sodium voltage-dependent channel; NPY, neuropeptide Y-expressing cells; NT, neurotransmitter; OPTO, optogenetics; P, pyramidal cells; PFC, prefrontal cortex; PV, parvalbumin-positive cells; SOM, somatostatin-positive cells; SWR, sharp-wave ripple.

Parvalbumin was the first calciprotein described in 1936 and purified in 1952 (Henrotte, 1952). The protein is organized in three domains: a 12 amino acid loop surrounded by two 8–9 amino acid alpha-helices, referred to as A, B, C, D, E, and F, according to its position to the *N*-terminus of the protein [Figure 1A(I); parvalbumin protein representation was adapted from https://www.uniprot.org/uniprot/P20472]. Generally, the EF-hands function in Ca^2+^ binding with rapid to intermediate kinetics and affinity (Schmidt, 2012). Although Ca^2+^ binding proteins constitute a large family of proteins with a high binding capacity for Ca^2+^, specific functions within an intricate network characterize many proteins and cellular mechanisms involved in Ca^2+^ signaling and Ca^2+^ buffering, an essential part of the Ca^2+^ homeostasis [Figure 1A(II)]. Specifically, their kinetics appear to differ, and parvalbumin is reported to exhibit slow-binding kinetics (Schwaller, 2009).

Parvalbumin binds Ca^2+^ and Mg^2+^ with affinities in the nanomolar and micromolar range, respectively (Haiech et al., 1979). It participates in a retro-control of the Ca^2+^ signal and, therefore, in the form of temporal regulation of Ca^2+^ homeostasis involved in accelerating the return to basal cytosolic Ca^2+^ concentrations in specific cells (Haiech et al., 2019). This unique kinetics possibly reflects the co-affinity of its Ca^2+^ binding sites for Mg^2+^, which needs to be displaced before Ca^2+^ -binding can occur (Schwaller, 2009).

The α-parvalbumin and β-parvalbumin (Oncomodulin) lineages, the two isoforms of parvalbumin expressed in mammals, exhibit markedly different Ca^2+^ and Mg^2+^-binding affinities, although they exhibit 49% homology in their structural amino acid sequence. Some amino acid differences in specific regions and structural alterations of these proteins result in significant differences in isoelectric point (pI < 5 for β), in the C-terminal helix length (usually with a longer residue in β), and properties of Ca^2+^ and Mg^2+^ binding and free energy change for divalent ions (Agah et al., 2003). Those changes in free energy for the binding of divalent ions would correspond to the difference in stability between unbound and bound forms, which is relevant for binding affinity (Agah et al., 2003).

Some evidence suggests that structural features outside the EF-hand motifs influence the affinity to divalent ions-binding in these two proteins. α-parvalbumin is unique in its high Ca^2+^ affinity relative to β-parvalbumin isoform, as it selectively binds Ca^2+^ over Mg^2+^ by ≈10 kcal/mol. A recent study indicates that, although the intrinsic characteristics of the EF-hand contribute strongly to the selectivity of Ca^2+^ in α-parvalbumin, allosteric changes affecting secondary and tertiary structures play a significant role in differentiating strong from weak Ca^2+^ binding. The authors also report that Ca^2+^ affinity and selectivity against Mg^2+^ are properties that emerge both from local effects at ion binding sites and non-local contributions elsewhere (Immadisetty et al., 2021). In addition to the morphofunctional characteristics of each parvalbumin isoform, its location is also an essential factor in the myriad of physiological processes regulated by these Ca^2+^/Mg^2+^ binding proteins (Schwaller, 2009). The α-parvalbumin isoform is found in skeletal muscles, GABAergic neurons, and the outer and inner hair cells of the cochlea. Although β-parvalbumin is restricted to the outer hair cells of the cochlea, it is expressed and secreted by macrophages and neutrophils, serving as a neuronal growth factor (Vologzhannikova et al., 2021).

Most of the GABAergic synaptic inhibition throughout the neocortex and hippocampal formation is thought to originate from a heterogeneous population of locally projecting interneurons (Ben-Ari et al., 2021), including parvalbumin-expressing cells (Tremblay et al., 2016). Neuronal network functions depend markedly on the signaling characteristics of GABAergic inhibitory neurons. Interneurons include a diverse population subdivided according to the morphologic and physiological properties, neurochemical marker content, and most notably, the location of their axon terminals [Figure 1A(III)].

When distant species (rodent, monkey, and human) are compared, some classic works emphasize the similarities of the expressions of parvalbumin and calbindin (Seress and Pokorny, 1981). However, in the work of Seress et al. (1993), differences were highlighted about the location, distribution, and organization of the main layers and PV+ interneurons, mainly between monkeys and humans. Although similar subpopulations in the three species have neurons that express parvalbumin or calbindin, the human hippocampus has more frequently neurons that contain calcium-binding proteins in both the molecular layer of the dentate gyrus and the layer of the stratum lacunosum-moleculare of the Ammon’s horn, unlike rodents and monkeys which, in the corresponding areas, present calbindin. However, despite differences in protein subpopulations and types, target selectivity did not change between species, as PV+ cells usually project to the somatic region and calbindin cells project to the most distal dendritic region of the principal cells (Seress et al., 1993).

PV+ interneurons are classified into two subgroups according to the synaptic input, the axo-axonic (chandelier) and the axo-somatic (basket cells) (Varga et al., 2014). Axo-axonic cells contact the initial axon segment of principal postsynaptic cells, whereas axo-somatic cells contact their target neurons’ soma and proximal dendrites. Either way, both subgroups promote accurate temporal inhibition (Freund and Buzsáki, 1998; Hu et al., 2014).

The morphology and characteristics of PV+ interneurons were elucidated in studies with different species, indicating that in primates, PV+ interneurons were found mainly in the hilus of the dentate gyrus, in the strata oriens layer, and the pyramidal layer of the Ammon’s horn. The predominant synapses were axo-somatic symmetric and asymmetric. Multipolar basket cell-like neurons in the neocortex were prominent in CA1. Also, in this subpopulation, it was identified that most dendrites have smooth, spineless dendrites or aspiny, with synapses occurring on the central axis of their dendrites, in addition to being postsynaptic to other axonal terminals (Ribak et al., 1993).

Findings also exist for more symmetric axo-somatic synapses in pyramidal cells in the region of CA2 than CA1 and CA3. Additionally, it is understood that the connection of PV+ interneurons dendrites occurs by gap junctions along the dentate gyrus and Ammon’s horn. Descriptions of the morphology, distribution, and location of such advanced neurons that hippocampal cells form a subset of GABAergic neurons, a local circuit, and that both feedback and feedforward inhibition participate (Soriano et al., 1990).

The GABA remarkably synchronized release is responsible for this temporal accuracy. Both precisions in the timing and high probability of GABA release are determined mainly by the time course of the presynaptic Ca^2+^ transient (Kraushaar and Jonas, 2000). The tight coupling of the Ca^2+^ source, the voltage-dependent Ca^2+^ channels (Hefft and Jonas, 2005), and the Ca^2+^ sensor (Bucurenciu et al., 2008) mediates the cytosolic Ca^2+^ concentrations in parvalbumin interneurons. Therefore, GABA spillover is evoked by a few presynaptic Ca^2+^ channels, which minimize asynchronous release and support phasic and precise output transmission (Bucurenciu et al., 2010). Thus, although parvalbumin exhibits slow binding kinetics compared to other calcium-binding proteins, the return to baseline cytosolic Ca^2+^ concentrations appears essential for the intrinsic timing accuracy of GABA release (Bartos and Elgueta, 2012).

Moreover, PV+ interneurons present additional electrophysiological properties that contribute to a fast and synchronous output. Such cells can discharge trains of short-duration action potentials at high frequencies (that can reach > 150 Hz at physiological temperatures) [Figure 1B(I)]. They may show a low input resistance somatodendritic and develop fast membrane time constant (Doischer et al., 2008; Norenberg et al., 2010) during adolescence/adulthood, implying a low membrane resistance that supports fast propagation of postsynaptic currents. Therefore, a fast-propagating postsynaptic current will arrive at the soma, defining a narrow time window for temporal summation (Bartos and Elgueta, 2012). Additionally, there is low voltage-dependent Na^+^ conductance at their dendrites [Figure 1B(II)], with a high density of voltage-dependent K^+^ [Figure 1B(III); Hu et al., 2010]. Interestingly, K^+^ conductance facilitates the decay time course of postsynaptic current at apical dendrites of these cells (rapidly and precisely reestablishing the resting potential), consistent with precise and reliable recruitment (Doischer et al., 2008; Hu et al., 2014).

Studies also revealed several remarkable properties of axons of hippocampal PV+ interneurons. In contrast, there is a low excitability somatodendritic domain and a highly excitable axonal, separated by a steep transition zone, represented by a stepwise increase in Na^+^ channels density (Hu et al., 2014). A high density of voltage-dependent K^+^ in the axonal segment is still observed, and the high activation threshold and the fast deactivation of these channels may ensure rapid action potential repolarization in the axon (Rudy and McBain, 2001; Hu et al., 2014).

Interneurons receive input from different afferents constituting feedforward and feedback pathways. In a feedback pathway, usually, there is reciprocal coupling between a fast-spiking parvalbumin interneuron and a principal cell (Hu et al., 2018). A feedforward inhibition is observed when a principal cell and an interneuron receive excitatory inputs from the same presynaptic source, and the interneuron then outputs its inhibitory signal to the principal cell. Thus, upon activation of the presynaptic source, the principal cell receives two input types, excitatory and inhibitory, separated by a brief delay (as parvalbumin action potential propagates fast), enabling interneuron integration (Ferrante et al., 2009). Therefore, this shift from inhibitory weight in dendrites toward a fast perisomatic inhibition may be a key mechanism in supporting high coherence network oscillations (Bartos and Elgueta, 2012). The activity of PV+ interneurons changes during network oscillations. In the absence of oscillatory activity, action potential frequency can be low (6.5 Hz) (Lapray et al., 2012). During theta oscillations, for instance, action potential frequency markedly increases in the hippocampus (21 Hz), and during sharp-wave ripples, the firing frequency increases by more than one order of magnitude (122 Hz) [Figure 1B(IV); Varga et al., 2012]. Sharp-wave ripples represent the most synchronous population pattern in the mammalian brain (Freund and Buzsáki, 1998). The synchronous output affects the entire brain, including a wide area of the cortex and several subcortical nuclei, characterizing “*off-line*” states of the brain, place coding, and complex behavioral repertoire [Hu et al., 2014; Buzsáki, 2015; Figure 1B(IV)].

Figure 1C shows a schematic representation of the circuit present in CA1 of the hippocampus with only a few connections, but other circuitry configurations from different hippocampal layers and different regions will be discussed in this Review and thus the corresponding circuitry singularities in these other regions will be addressed in the text.

The balance on inhibition/excitation can be crucial since multiple inhibitory mechanisms finely regulate excitability [Figures 1C(I,II)]. Therefore, alterations in feedforward inhibition can lead to epileptic activity (Sloviter, 1991) and the propagation of epileptiform waves (Trevelyan et al., 2007) (Figures 1C(III–V)]. Spiking activity of perisomatic targeting interneurons under the right circumstances can facilitate the generation of sharp-wave ripples, and in the wake of inhibition, rebound synchronization of a critical number of pyramidal neurons may ignite a population burst (Ellender et al., 2010). Still, regions of eroded inhibition may present fast ripples at the onset of some types of focal seizures, representing a hypersynchronous interneuronal network and/or associated with out-of-phase pattern (Jiruska et al., 2017).

Also, it has been an emerging consensus that parvalbumin neurons are more susceptible to degeneration (Fairless et al., 2019). The ability to efficiently buffer Ca^2+^ since active extrusion of this ion places a high metabolic demand on the cell adds to the significant amounts of energy needed to reestablish electrochemical gradients after action potentials and synaptic release (Magistretti and Allaman, 2015). Multiple injury pathways converge to an excessive rise in intracellular Ca^2+^ levels, activating proteolytic enzyme cascades, such as calpains and caspases, thus leading to the apoptosis pathway (Fairless et al., 2019). Therefore, maintaining calcium homeostasis within neurons is essential to sustain normal function, involving several mechanisms (Fairless et al., 2019).

## Parvalbumin Role in Epilepsy: Historical Investigation of Temporal Lobe Epilepsy in Clinical and Experimental Studies

Temporal lobe epilepsy (TLE) is the most common type of epilepsy in adult patients and one of the most challenging types for seizure control, with more than 30% of pharmacoresistance (Brodie and Dichter, 1996). In order to pave new avenues for the treatment, it is fundamental to improve the understanding of the epileptogenic process and the comorbidities associated with epilepsies. Preclinical studies have contributed to enormous advances by dissecting the main pathophysiological characteristics of epilepsies. The cross-validation of animal and human findings adds considerable value to epilepsy research because it contributes to the deeper understanding of the mechanisms of epileptogenesis and ictogenesis (Pitkänen and Engel, 2014; Devinsky et al., 2018).

The seminal studies of Sloviter evaluated the hypothesis that there are selective changes of specific interneuronal cell types, which may be related to differences between cell populations in their abilities to buffer intracellular calcium (Sloviter, 1989). The quantification of both the density and staining intensity of calcium-binding protein and GABA interneurons in sclerotic hippocampi (specifically surviving hilar neurons) compared to autopsy controls or hippocampi from tumor-associated patient group suggest that calcium-binding protein synthesis was increased (Sloviter et al., 1991) (although it was discussed GABAergic neuron densities *per se* are not necessarily related to either hippocampal sclerosis) (Babb et al., 1989). Does surviving calcium-binding protein-containing cells represent a protective/surviving process (as noticed by their increase) or does the loss of these neurons and their particular innervation suggest that they are intrinsically related to the pathophysiology? (Sloviter, 1991).

Pivotal studies report findings showed increased reactive synaptogenesis of fibers labeled for GABAergic interneurons in resected epileptogenic tissue. Also, it was observed preferential losses of hippocampal/CA4 hilar neurons from extrahippocampal mass lesions or idiopathic patients. Those mapped GABAergic neurons were especially basket cells and hilar interneurons, which are involved in several feedforward and feedback axon circuits onto principal cells and other interneurons (Mathern et al., 1995). Some of these findings were previously observed in experimental models (Marx et al., 2013) that could present 71 and 97% in the hippocampus of cell loss in the pilocarpine model (Best et al., 1993, 1994) and affect subiculum and entorhinal cortex in presubicular and parasubicular layers (Drexel et al., 2011).

Although parvalbumin immunoreactive cells are only a subset of GABA and calcium-binding neurons, a loss of parvalbumin could mean an essential and specific loss of a subpopulation of inhibitory neurons. However, several studies pointed that the observed reduction in parvalbumin- immunoreactivity could be associated with the parvalbumin protein expression levels and not the loss of the parvalbumin-expressing GABAergic neurons (Filice et al., 2016; Medici et al., 2016). The reduction of GABAergic and parvalbumin staining of the axo-somatic plexus in the hippocampal granule cell layer has been observed in clinical studies, further confirmed in experimental epilepsy models [Sloviter, 1991; Zhu et al., 1997; Kobayashi and Buckmaster, 2003; Arellano et al., 2004; Van Vliet et al., 2004; Figures 1C(III,IV)].

Analysis of postsynaptic target elements of PV+ axon terminals showed that they form symmetric synapses with soma, dendrites, axon initial segments, and spines as in control, but the ratio of axon initial segment synapses was increased in the epileptic tissue. Furthermore, the inputs in initial axon segments increased about three times in the epileptic samples (Wittner et al., 2001). At the same time, a somatodendritic compartment was observed, and partly the end axon losses of parvalbumin (Maglóczky and Freund, 1995; Scotti et al., 1997). Thus, researchers reported that, despite a simple loss or increase in parvalbumin number/immunoreactivity in hippocampal dentate/hilar subregion, there is a local unbalanced innervation, mostly axo-axonic synapse into granule cells, which could be the key finding in epileptogenesis (Wittner et al., 2001; Arellano et al., 2004; Andrioli et al., 2007).

So, additionally to the change of the specific above-mentioned local-circuit neurons, parvalbumin could also be responsible for synaptic reorganization in epilepsy by forming abnormal synapses. A recent finding demonstrates that in the resected sclerotic hippocampus of TLE patients, the sprouting of parvalbumin axons in dendrites and spines of granule cells in the dentate molecular layer was observed with the presence of ectopic PV+ neurons [Ábrahám et al., 2020; Figure 1C(V)]. The abnormal presence may also be found in other hippocampal layers. In the CA1 region, PV+ cells and axons were found only in non-sclerotic cases (Wittner et al., 2005). Also in a report with TLE patients hippocampal/entorhinal cortex, single-unit deep electrode monitoring revealed that interneuronal firing during seizure generation and spread spawns a subsequent increase in excitatory neuron firing and seizure evolution (Elahian et al., 2018).

Parvalbumin innervation appears to be restrictedly evaluated in other regions than the hippocampus. Neuronal cell densities in the basolateral complex of the amygdala were significantly reduced in the lateral nucleus (LA) in TLE patients as compared to the autopsy, followed by a parvalbumin reduction. Ultrastructure analysis revealed a reduction in perisomatic boutons and a remarkable reduction of axo-somatic GABAergic input onto excitatory cells, which correlated with a higher perisomatic fibrillary gliosis (Yilmazer-Hanke et al., 2007). Also, there are reports showing abnormal parvalbumin morphology in focal cortical dysplasia (FCD). In non-dysplastic neocortical control tissue, calbindin- and PV+ cells were confined primarily to neocortical layers II/III tissue, whereas tissue from patients with FCD showed an abnormal distribution of these cells throughout all cortical layers; some areas contained small clusters of interneurons, whereas, in many regions, calbindin- and PV+ cells were scarce (Calcagnotto et al., 2005).

Therefore, this brief overview of the histological and ultrastructure investigation of parvalbumin in human hippocampal epilepsy is not a simple change in the number of this interneuronal subpopulation. Instead, there is probably morphological plasticity in which increases in the initial axon segments and/or reductions or abnormal dendritic and somatic innervations contribute to excitability and synchronization (for further revision on the complexity of GABAergic interneuronal role in epilepsy check Marafiga et al., 2021).

The appropriate use of animal models is fundamental since clinical studies cannot fully access parvalbumin and its mechanical comprehension in epilepsy. The most frequent rodent models used to generate spontaneous recurrent seizures can be divided into three main groups: chemical, electrical, or genetic models (Kandratavicius et al., 2014). Seizure models induced by chemical convulsants other than kainic acid, pilocarpine, such as bicuculline or pentylenetetrazol (PTZ), are helpful for several purposes in screening for new antiepileptic drugs (Löscher and Lo, 2011). In turn, models generated by electric stimulation have the advantage of reproducing core features of epileptogenesis in specific brain regions with low mortality, which can be realized by single-evoked epileptic after discharges or chronic stimulations with a progressive enhancement of seizures susceptibility (Rolston et al., 2011). Lastly, several genetic models are used to test hypotheses involving specific mutations and their specific contributory role to seizure generation/propagation; however, the underlying genetic alterations in epilepsy remain not fully understood (Gonzalez-Sulser, 2020). Several conditional genetic manipulations allow the modification of parvalbumin-expressing neurons (Jiang et al., 2018; Malik et al., 2019; Sharma et al., 2021).

This review grouped additionally to the clinical findings, the main experimental models employed in epilepsy research, including seizure models induced by chemical seizures, seizures induced by electrical stimulation, and genetic manipulations. Some of the most known types of epilepsy and their corresponding experimental models will be discussed, integrating the role of parvalbumin in seizure generation and propagation in a translational fashion.

### Parvalbumin in Other Chemical and Electrical Experimental Models

The TLE condition, as previously discussed, was extensively studied in experimental models such as pilocarpine and kainate, which made significant contributions on parvalbumin role in epilepsy, together with the clinical findings (Leite et al., 2002). The surviving interneurons that show those extensive changes in morphology, layer-specific loss of expression also present subcellular distribution of GABA_*A*_ receptor subunits (Loup et al., 2000). It is known that GABAergic interneurons are essential to generate and promote synchronized activity and coherence of oscillations (Galarreta and Hestrin, 2002), especially in the parvalbumin cells, facilitating current reversal in the presence of high-frequency stimulation (Schwaller et al., 2004). Therefore, drugs that allosterically modulate the GABA_*A*_ receptor channels, such as antagonists, can act on GABAergic neurotransmission in a proconvulsant manner (Olsen, 2018). The evaluation of seizures through inhibitory neurotransmission can be performed with bicuculline that blocks GABA_*A*_ receptors and modifies the cellular organization of the parvalbumin neurons subpopulation, reducing the number of these neurons in the neostriatum in rats exposed to bicuculline in early life (Luk and Sadikot, 2001). PTZ is also another chemical model that was deeply investigated as an epilepsy experimental model. In addition to PTZ being a strategy for generating seizures, it can be used in kindling protocols, in which it was also demonstrated there is a loss of parvalbumin neurons (Ueno et al., 2019).

Electrical models usually stimulate specific areas with the exception of maximal electroshock (MES). However, a model based entirely on the specific electrical manipulation of PV+ interneurons is not yet possible, making the association of genetic and optogenetic techniques necessary. Nevertheless, it is possible to stimulate areas with an intense concentration of PV+ interneurons, such as the prefrontal cortex (Bjerke et al., 2021), since GABAergic interneurons present in all neocortical areas are characterized by the expression of three distinct classes of largely non-overlapping Ca^2+^-binding proteins, which are parvalbumin, somatostatin, and the ionotropic serotonin receptor 5HT_3A_ (Tremblay et al., 2016).

### Genetic Factors: Receptor and Ion Channel Mutations, Gain and Loss of Function, and Changes in Parvalbumin Developmental Trajectories in Epilepsy

While in the etiology of most epilepsies, a combination of both acquired and genetic factors is involved, epilepsies with a determinant genetic background (known as idiopathic) constitute only a minority of all seizure disorders (Andrade and Minassian, 2007) being estimated to account for ∼15–20% (Kang, 2017). The latter, nevertheless, provide an important source for our increasing knowledge about the genes that can be involved in epileptogenesis and help us gain insight into the mechanisms underlying the forms of epilepsy (Steinlein, 2008).

Dysfunctions of mutated voltage- or ligand-gated ion channels have been considered to be a significant cause of idiopathic epilepsies (Steinlein, 2008; Lerche et al., 2013), which was even considered for many years as synonymous. However, new findings indicate that mutations in many non-ion channel genes have a detrimental role in seizure generation and propagation (Kang, 2017). In genetic epilepsies, it is usually observed ion channels gain or loss of function, many of which may affect GABAergic signaling through alterations of PV+ interneurons’ function and will be further explored in this review.

Genes encoding subunits of the ionotropic GABA_*A*_ receptors and metabotropic GABA_*B*_ receptors are also significant candidates for involvement in epilepsy, considering the central role of GABA in mediating inhibition in the brain. Mutations in two of these genes have been associated with epilepsy in humans (Burgess, 2006), but most known are the channelopathies. Lastly, changes in the development trajectory of interneuronal cells may profoundly impact brain function that can lead to different seizure phenotypes. There is a vast diversity of mutations, knockouts, and other genetic manipulations in the GABAergic system that produce an even more significant phenotypic heterogeneity of epilepsy outcomes. For this review, we will attain reports that evaluate parvalbumin interplay.

Although several studies demonstrated that GABA receptor-mediated inhibition could actively contribute to epileptiform interictal oscillations (seizures induced by applying K^+^ channel blockers and blocked by GABA_*A*_ antagonists – for review see Avoli, 2019), only a few studies have highlighted the parvalbumin direct and specificity changes related to the genes that code GABA receptor subunits (mostly GABA_*A*_) and are associated with epilepsies (MacDonald et al., 2012; Pitkänen et al., 2016; Kang, 2017). GABA receptor mutation, large deletions, and/or changes in translocations have been associated with childhood absence epilepsy, febrile seizures, epileptic encephalopathy, absence epilepsy diversity of myoclonic epilepsy and generalized tonic-clonic seizures, Dravet Syndrome, to catastrophic seizure phenotypes that may lead to death very early in life (Steinlein, 2008; Kang, 2017). While a close link between GABA receptor genes and PV+ interneurons is hard to trace, voltage Na^+^-gated sodium (Na_*v*_) is attributed mainly to parvalbumin dysfunction (Yu et al., 2006; Ogiwara et al., 2007; Yamakawa, 2011; Baraban et al., 2013; Jiang et al., 2016; Schutte et al., 2016). Na_*v*_ channels are essential for neuronal excitability because they transiently increase the membrane conductance to Na^+^ in response to depolarization, initiating potential action generation. Na_*v*_ channels in the brain are formed by a principal α subunit (Na_*v*_ 1.1 to Na_*v*_ 1.9, coded by the genes *SCN1A* to *SCN11A*). Na_*v*_ channels have been implicated in numerous neurologic diseases, and Na_*v*_ 1.1/*SCN1A* is a significant target of epileptogenic mutations. Thus, the reduced firing of inhibitory neurons through sodium channel loss of function may affect GABA release (Mantegazza and Broccoli, 2019). Several studies support that the axonal Na_*v*_ 1.1 localization is primarily localized in parvalbumin interneuron axons (except for the somata of hippocampal non-pyramidal cells). It is suggested that Na_*v*_ 1.1 is involved in the maintenance but not in the initiation of sustained fast spiking in the interneurons and probably also in regulating GABA release from the interneurons (Yu et al., 2006; Ogiwara et al., 2007). The critical mechanism of interneuron dysfunction was a deficit of action potential initiation at the initial axon segment, which increased with the duration of firing periods, suggesting that increased slow inactivation could also play an important role. The deficit in interneuron firing reduces action potential-driven inhibition of excitatory neurons as revealed by less frequent spontaneous inhibitory postsynaptic currents (IPSC) (Hedrich et al., 2014). Since the discovery of techniques to manipulate gene expression, Na_*v*_ 1.1 mouse models that evaluate loss-of-function mutations have investigated the role of this channel in PV+ interneurons in epileptic phenotypes. Those studies confirmed that this deficiency in Na_*v*_ 1.1 is responsible for the collapse of action potentials at higher firing frequencies in inhibitory neurons (Yu et al., 2006). Generally, the heterozygous deletion exhibits temperature-induced and spontaneous seizures, mild ataxia, premature death, and sufficient to cause a Dravet-like phenotype (Cheah et al., 2012; Richards et al., 2018). These data provide evidence that the inability of GABAergic interneurons to fire robustly results in hyperexcitability, leading to seizures.

A recent paper investigated the role of parvalbumin in the synchronization and temperature-induced seizure model of Dravet syndrome in parvalbumin-*Scn1a*^+/–^ mice (male and female) using *in vivo* two-photon calcium imaging in the neocortex (Tran et al., 2020). It was observed that wild-type parvalbumin-*Scn1a* mice showed a progressive synchronization in response to temperature elevation, which is absent in parvalbumin-*Scn1a*^+/–^ mice (Tran et al., 2020). Those mice showed higher activity of both putative principal cells and parvalbumin cells (Tran et al., 2020). Interestingly, the authors further discussed the previously known results of parvalbumin-*Scn1a*^+/–^ mice that can survive beyond adolescence, recover normal intrinsic excitability supporting the conclusion that parvalbumin excitability normalizes over-development (Tran et al., 2020).

A report showed that another class of channel, the voltage-dependent calcium (Ca_*v*_) 2.1 (P/Q-type), could also be of great importance in sustaining parvalbumin dynamics and could be implicated in the epileptogenic process. Researchers developed an exciting new model using a conditional genetic approach to selectively ablate *CACNA1A* in specific subsets of cortical GABAergic interneurons. The regionally knockout animals exhibit a severe form of generalized epilepsy. It was also demonstrated that this mutation selectively impaired GABA release from parvalbumin, also leading to unreliable transmission with high failure rates and perturbed kinetics (Rossignol et al., 2013). As mentioned previously, there are more than a dozen variations of genes that encode the principal subunits of voltage channels which can be implicated in genetic epilepsies. Future studies might reveal that some of those genes could be also expressed in parvalbumin neurons and may be considered the target of similar studies.

Additionally, as previously highlighted, one must also consider the genetic regulation of inhibitory interneurons during development. Influences of environment timed programmed patterns and epigenetic interplay has been argued to have a detrimental role in the genetic background of epilepsies. During early development, ionotropic GABA receptors mediate depolarizing currents, which activate calcium-sensitive signaling processes that are vital for neuronal differentiation and brain development (Galanopoulou, 2008). For instance, the most notorious mechanism of changes in maturation of inhibitory neurons involves the cation-chloride cotransporters (CCC) that can involve potassium (KCC) and both sodium and potassium (NKCC2). CCC is in a critical position to control and coordinate the development of GABAergic transmission. It is not surprising that CCC dysfunctions are likely to be associated with a wide range of neurological and psychiatric disorders (Kaila et al., 2014; Puskarjov et al., 2014). Changes in gene transcription regulation that specify parvalbumin fate and identity, including differentiation, migration, surrounding perineural network, and development, can reduce cortical parvalbumin cells, altered morphology, and immature electrophysiological properties firing rates and lower power of gamma oscillations (Reh et al., 2020).

The mammalian target of rapamycin (mTOR) regulates several cellular processes and death cascades by regulating mRNA translation (Takei and Nawa, 2014). The mTOR pathway has been implicated as a mechanism by which diverse genetic mutations and acquired abnormalities lead to a final common pathway of seizures (Griffith and Wong, 2018). The 4E-BP2 is the major neuronal mTORC1-downstream and is a translational repressor, which inhibits cap-dependent translation (Nguyen and Bordey, 2021). The ablation of 4E-BP2 in PV+ interneurons, but not in other subtypes, is sufficient to promote reduced latency and increased severity to PTZ-induced seizures. These changes in 4E-BP2 deleted mice were followed by a reduction in PV+ interneurons number in the adult hippocampus which could also contribute to the epileptogenesis process (Sharma et al., 2021).

Mutations in several transcription factors have been described to play a role in epilepsy (for further revision, please refer to Powell, 2013; Jiang et al., 2016; Gilsoul et al., 2019). Animal models carrying those gene variants were generated, providing unique discoveries for understanding the role of parvalbumin in epilepsy. Also, some of them exhibit autism-related behaviors associated with seizure activity, which represents an important phenotype for studying these comorbidities (Peñagarikano et al., 2011; Bridi et al., 2017).

In this regard, the knockout genetic models have indicated that a single alteration of PV+ interneurons plays a fundamental role in epileptiform activity after a second hit. Selective interneuron ablation (injection of *Gad2-ires-Cre* with an adeno-associated virus containing the diphtheria toxin receptor) consistently caused SRSs (not SE) but did not persisted (Spampanato and Dudek, 2017). Parvalbumin^–/–^ mice do not present significant abnormalities during development, but the severity of seizures induced by PTZ is significantly greater than parvalbumin^+/+^ subjects (Schwaller et al., 2004). Additionally, *in vivo* extracellular single-unit activity shows an increase of units regularly firing in the temporal cortex of parvalbumin^–/–^ mice while burst firing decreases. Schwaller et al. (2004) propose that the firing pattern shift increased the probability of synchronous firing, which increased the epileptic susceptibility in parvalbumin^–/–^ mice. However, intrahippocampal kainic acid (KA) injection does not increase neurodegenerative and morphogenic effects in parvalbumin^–/–^ mice, indicating that KA effects are not altered in the absence of parvalbumin alone (Bouilleret et al., 2000). Another study demonstrated that in KA-induced seizures in mice with parvalbumin deficiency there is a facilitation of postsynaptic inhibition currents (IPCSs) and gamma oscillations in the hippocampus (Vreugdenhil et al., 2003). Besides, the selective elimination of muscarinic acetylcholine M1 receptors in PV+ interneurons prevented pilocarpine-induced excitation and reduced the severity of seizures (Yi et al., 2015). These discrepancies may indicate different participation of PV+ interneurons considering the second hit as different chemical models.

### Genetic Factors: Multigenic Variants in Genetic Strains

Genetic models of epilepsy have long been used to study network phenomena underlying particular forms of epilepsy. For instance, many selected strains carrying multigenic variants were also important to understand the role of parvalbumin in epilepsies. Many of the described epilepsy-prone strains have mapped gene expression of GABA receptors and voltage channels related to parvalbumin, but some animal models have not established a direct link with this interneuronal specific sub-class.

In the genetically epilepsy-prone hamster (GPG/Vall), parvalbumin was evaluated in the central auditory neurons. Cochlea and other auditory nuclei showed decreased parvalbumin volume and cell size at the same time it exhibited greater density. The authors interpreted this change in number and morphology as a protective mechanism to prevent cell death in the face of reduced afferent input (Fuentes-Santamaría et al., 2005).

Wistar Audiogenic Rats (WAR) is a genetically selected seizure-prone rat strain susceptible to audiogenic seizures when exposed to high-intensity acoustic stimulation (Garcia-Cairasco et al., 2017). These audiogenic-like seizures can be altered by GABAergic agonist or antagonist injection in the colliculus as well as deafferentation, demonstrating WARs show GABAergic deficiency in the midbrain, with specific changes in the posterior superior colliculi. Therefore, the group sustains that the inhibitory changes in auditory nuclei could play a contributory role to the audiogenic seizure activity in WARs (Terra and Garcia-Cairasco, 1992; Tsutsui et al., 1992; Garcia-Cairasco et al., 1993). Additionally, impaired GABAergic modulation in the CA1 region of the hippocampus (Rossetti et al., 2011) and a functional reduction of GABAergic neurotransmission in hippocampal slices from WARs were detected (Drumond et al., 2011). Recently, the group has demonstrated that even in the absence of previous seizures, GABAergic inhibition toward CA1 pyramidal neurons is reduced in WARs. Miniature Inhibitory Postsynaptic Currents (mIPSPs) are faster and less frequent in WARs, pointing to a particular change in the kinetics of mIPSPs. Whereas fast rise times are kept, longer rise times are altered. It is proposed that while some fast kinetic subpopulation is kept, the slower subpopulation might be lost and inhibitory neurons with a peak close to 1.4 ms are enhanced (Cunha et al., 2018). This peak is similar to feedback and feedforward interneurons which could be parvalbumin- and cholecystokinin-containing (CCK) basket cells (Elfant et al., 2008). Authors observed that consequently to the longer rise times altered, mIPSCs in WAR were separated by longer inter-event intervals, which could also reflect a change in the number of active synapses, release probability, or input location (Cunha et al., 2018). It is discussed that WAR deficiency in both midbrain and hippocampus interneural inhibitory input could contribute to the seizure-dependent generation and spread of hyperexcitation in those seizure-prone animals, and would be interesting to evaluate if PV+ interneurons are related to seizure vulnerability and prosencephalic recruitment.

In the Wistar Albino Glaxo from Rijswijk (WAG/Rij) rat, a genetic model of absence epilepsy, the somatosensory cortex contains a focus that initiates a cascade of events that ultimately leads to the occurrence of the bilateral and generalized SWDs (van Luijtelaar and Sitnikova, 2006; Bazyan and van Luijtelaar, 2013). Quantification of PV+ interneurons showed a deficient global (parvalbumin) and local GABAergic (neurophysiological) system in the neocortex, which may explain why specifically the perioral region of the somatosensory cortex is hyperexcitable and the 10 Hz oscillations in the initiation site (van Luijtelaar and Sitnikova, 2006). It was also noted a deficiency in the expression of genes coding for the low threshold T-type Ca^2+^ channel, lower levels of Ca^2+^-binding protein in these corresponding structures (Bazyan and van Luijtelaar, 2013).

The stargazer mouse model is another model of absence epilepsy in which administration of a competitive NMDA receptor antagonist markedly exacerbates seizures. This strain carries a mutation in stargazin, an AMPA receptor trafficking protein. It was observed that in stargazer animals, AMPA receptor localization is detected exclusively in PV+ fast-spiking interneurons in the somatosensory cortex. PV+ cortical interneurons in stargazers show a near twofold decrease in the dendrite: soma Ca^2+^-permeable AMPA receptor subunit expression ratio, indicating that hyperexcitability induced by NMDA receptor modulation was mediated through interneurons (Maheshwari et al., 2013). Loss of synaptic AMPAR-mediated excitation of cortical PV+ inhibitory neurons likely impairs feedforward inhibitory output and contributes to the generation of SWDs and absence seizures in stargazers (Adotevi and Leitch, 2017). Again, the paradoxical excitability could be related to the interneuron-dependent mechanism for activation, and balance between excitation/inhibition.

## Psychiatric Comorbidities in Epilepsy

The psychiatric comorbidities in epilepsy are frequent and have a significant impact on the life quality of patients (Hermann et al., 2000; Johnson et al., 2004; Kanner et al., 2018). In epileptic patients, the lifetime prevalence of psychiatric comorbidities can reach up to 48% in some studies (Jalava and Sillanpää, 1996; Gaitatzis et al., 2004; Burneo et al., 2005). The prevalence of psychiatric disorders may also differ according to the type of epilepsy, as the risk in patients with TLE is 60%, in focal epilepsy is 54%, and in patients with primary generalized epilepsy is 37% (Edeh and Toone, 1987). Among the most common psychiatric disorders in epilepsy, major depressive disorder, anxiety, and psychosis, which neuropathological mechanisms may be associated with PV+ interneurons activity (Salpekar and Mula, 2018; Figure 1D).

### Major Depressive Disorder

Major depressive disorder is the most common psychiatric comorbidity in epilepsy, with a lifetime prevalence of 6–30% and up to 50% in patients with recurrent seizures (Kanner, 2003; Tellez-Zenteno et al., 2007). Depression in epilepsy is characterized by several emotional-cognitive alterations, which can lead to functional incapacity of the patient and chronically aggravate the seizures (Fleck et al., 2003; Adelöw et al., 2012). Epileptic patients with depression are 25% more likely to commit suicide and two times more likely to be pharmacoresistant (Hitiris et al., 2007; Tellez-Zenteno et al., 2007). In addition, evidence shows that epilepsy and depression have bidirectional relationships (Kanner, 2012; Kanner et al., 2018). Depressed patients increase 4-6 times the risk of epilepsy development, and stress is commonly associated with depressive symptoms and the precipitation of seizures (Hesdorffer et al., 2000, 2006; Nakken et al., 2005). In turn, treatment with antidepressants can decrease both depressive symptoms and the incidence of seizures (Kühn et al., 2003; Specchio et al., 2004; Alper et al., 2007; Kondziella and Asztely, 2009).

Despite the high prevalence and functional impairment of epilepsy associated with depression, the neurobiological mechanisms remain poorly understood (Kanner, 2005; Kanner et al., 2014). Among the different possibilities of pathogenic mechanisms common to both diseases, we can highlight the excitatory/inhibitory imbalance in limbic circuits (Valente and Busatto Filho, 2013; Shetty, 2014). The prevalence of depressive epileptic patients with seizures in temporal or frontal circuits is 55% (Jackson and Turkington, 2005). Also, depression in epileptic patients is associated with dysfunctional metabolism in the frontal and temporal lobe, which can even sustain or trigger seizures (Jokeit et al., 1997; Gilliam and Kanner, 2002; Lanteaume et al., 2009). In these limbic circuits affected by depression and epilepsy, the activity of PV+ interneurons may play a fundamental role in the regulation of excitatory/inhibitory imbalance (Shetty, 2014; Jiang et al., 2016; Yilmazer-Hanke et al., 2016).

Regarding depression, the main changes in the post-mortem analysis of PV+ interneurons occur in the prefrontal cortex, exhibiting a reduction in density (Khundakar et al., 2011). On the other hand, studies have shown in the prefrontal cortex of depressed patients no reduction of PV+ interneurons, despite the positive correlation between density PV+ interneurons and indications of metabolic disturbance in glutamate levels, usually associated with depressive symptoms (Cotter et al., 2002; Rajkowska et al., 2007).

To our knowledge, there are no studies that have directly investigated the relationship of PV+ interneurons in depressed epileptic patients. However, this relationship can be assessed using depressive-like behavioral tests such as the forced swim [Figure 1D(I)] and sucrose consumption tests in rodent epileptic models (Mazarati et al., 2009). Moreover, work from Csabai et al. (2017) investigated how chronic stress affects perisomatic inhibitory neurons and their synapses in the hippocampus of rodents can give us tips about neural working. In this study, they found a decrease in the density of PV+ interneurons, as opposed to cholecystokinin immunoreactive neurons, which showed no change in cell density. However, although a reduction of PV+ interneurons was observed, the perisomatic inhibitory synapses on CA1 pyramidal cells were unaffected by exposure to stress that induces a depressive-like behavior, in addition to not following apoptotic or necrotic processes, data that conflict with the remodeling of excitatory synapses in chronic stress models.

Animals submitted to amygdala kindling decrease long-term potentiation (LTP) in the amygdala and the hippocampus (Schubert et al., 2005). These effects are not restricted to the amygdala, and the stimulus propagation reaches the prefrontal cortex in the early kindling stages (Fernández-Mas et al., 1992). In a bidirectional manner, stress factors, such as corticosterone and psychological stress, can also decrease seizures threshold (Taher et al., 2005; Mazarati et al., 2009), as acute stress facilitates epileptic afterdischarges in the hippocampus, while chronic stress suppresses hippocampal LTP (Pavlides et al., 2002). The impairments of hippocampal PV+ interneurons seem to play a fundamental role in depressive-like behaviors associated with epileptic models. The epileptic hippocampus chronically decreased the density of PV+ interneurons, which is sufficient to activate the HPA axis (Earnheart et al., 2007; Schloesser et al., 2009; Hu et al., 2010). In turn, epileptic models with depressive-like behaviors present plasma corticosterone levels enhanced (Mazarati et al., 2009). Finally, chronic stress can amplify seizures since it reduces hippocampal PV+ interneurons (Hu et al., 2010; Czéh et al., 2015). The depressive-like behaviors can be prevented by antidepressants that revert the reduction of hippocampal and prefrontal PV+ interneurons (Filipović et al., 2018; Todorović et al., 2019).

Impairments of cortical PV+ interneurons are sufficient to induce generalized seizures (Rossignol et al., 2013), associated with deficits in cognition tasks and depressive-like behaviors (Bissonette et al., 2014). Selective suppression of PV+ interneurons in the prefrontal cortex using designer receptors exclusively activated by designer drugs (DREADD) promotes depressive-like behaviors [Figure 1 D(II)]. In contrast, selective chemogenetic excitation of PV+ interneurons can revert deficits and prefrontal plasticity disruptions (Perova et al., 2015). The optogenetic stimulation of PV+ interneurons in the hippocampus can also reduce seizure duration (Krook-Magnuson et al., 2013). Interestingly, stimulation of hippocampal parvalbumin-interneurons did not affect immobility in depressive behaviors (Zou et al., 2016). Despite the clinical and experimental evidence of the relationship between epilepsy, depression, and PV+ interneurons, further works are still necessary to directly investigate the causal relationship of these factors.

### Anxiety

The prevalence of anxiety in epilepsy in population-based studies is 13–23% (Brandt and Mula, 2016). Several authors consider anxiety the most underdiagnosed psychiatric comorbidity in epilepsy because it is commonly considered a natural consequence of seizures (Salpekar and Mula, 2018). However, epileptic patients are two times more likely to present anxiety disorders (Kobau et al., 2008; Ottman et al., 2011; Rai et al., 2012). Moreover, anxiety symptoms aggravate the side effects of antiepileptic drugs, increase the severity of epilepsy and impairs memory tasks in epileptic patients (Beyenburg et al., 2005; Gómez-Arias et al., 2012; Jacoby et al., 2015; Mula, 2016). Similar to depression in epilepsy, previous history of anxiety disorders increased almost three times the risk of developing seizures, and it is significantly associated with focal epilepsy (Kimiskidis et al., 2007). In epilepsy, the comorbidities of depression and anxiety are common; epileptic patients with depression, which also experienced anxiety disorders in their lifetime, represent up to 70%. Conversely, depression and anxiety share similar pharmacological treatments (Kanner et al., 2004; Kanner, 2009).

The putative neurobiological substrates of these psychiatric comorbidities remain obscure, but specifically to anxiety associated with epilepsy, amygdalar impairments of excitatory/inhibitory balance seem to be central (Aroniadou-Anderjaska et al., 2007). The incidence of anxiety is more frequent in pharmacoresistant TLE patients (Pham et al., 2017). Furthermore, patients with TLE show hyperexcitability in the amygdala, as well as anxiety disorders (Pitman et al., 2001; Nutt and Malizia, 2004; Aroniadou-Anderjaska et al., 2008). In neuroimaging studies, epileptic patients drug-resistant with anxiety show an enlargement of the amygdala (Satishchandra et al., 2003). However, other limbic structures such as the prefrontal cortex seem to be involved in anxiety (Pope et al., 2019). Epileptic patients with foci in the frontal areas show anxiety symptoms as well (Tang et al., 2012). Similar to depression, limbic circuits may play a key role in the neurobiology of anxiety symptoms in epileptic patients (Brandt and Mula, 2016).

In animal models, the antiepileptic effects of benzodiazepines also treat anxiety-like behaviors [Figure 1D(III); Mula et al., 2007]. In a genetic model of human generalized epilepsy, rats exhibited anxiety-like behaviors, such as elevated plus maze and open field arena, both before and after the onset of epilepsy, indicating a bidirectional relationship between anxiety and epilepsy (Jones et al., 2008). Early life status epilepticus induced by the lithium-pilocarpine model also increased anxiety-like behaviors in the elevated plus-maze (Loss et al., 2012). Even sub-convulsant doses of pilocarpine can lead to behavioral impairments in the elevated plus-maze and open field arena (Duarte et al., 2013). In the pilocarpine model, epileptic mice show decreased PV+ interneurons in the hippocampus, and anxiogenic behaviors were prevented and induced by GABA receptor agonists and antagonists, respectively (Zhu et al., 2019). Conversely, lesions in the ventral hippocampus reduced anxiety-like behaviors, while dorsal hippocampus stimulation-induced anxious behaviors (Kjelstrup et al., 2002; Adhikari et al., 2010, 2011; Weeden et al., 2015). In turn, anxiolytic drugs such as diazepam can also modulate PV+ interneurons density in the amygdala and hippocampus (Hale et al., 2010; Ravenelle et al., 2014). The anxiety-like behaviors and the number of PV+ neurons also significantly increased in the amygdala after the enriched environment, which positively correlates with each other (Urakawa et al., 2013). Finally, specific modulation of PV+ interneurons in the dentate gyrus by DREADD induced anxiolytic effects, without affecting depressive or psychotic-like behaviors [Zou et al., 2016; Figure 1D(III)].

### Psychosis

*Psychosis* is the mainly psychiatric comorbidity related to PV+ interneurons (Lodge et al., 2009; Cifelli and Grace, 2012). The life-prevalence of psychosis is 2–7% in epileptic patients and 6–12% in TLE, corresponding to the third psychiatric comorbidity more common in epilepsy (Gaitatzis et al., 2004; Kanner et al., 2004; Hippenmeyer et al., 2005; Clancy et al., 2014). While depression and anxiety in epilepsy seem to share common mechanisms, there is no consensus in the literature regarding the relationship between psychosis in epilepsy. Some authors suggest an antagonistic relationship between the neurobiological mechanisms of psychosis and epilepsy, while others indicate a similar mechanism (Kawakami and Itoh, 2017; Nakahara et al., 2018). It can be cited as evidence of antagonistic circuits between epilepsy and psychosis: seizure suppression and normalization of the EEG through anticonvulsants may lead to the emergence of psychotic symptoms (Kandratavicius et al., 2012); electroconvulsive therapy treat psychosis (Pollock, 1987); antipsychotic pharmacological therapy through dopamine antagonism might trigger seizures, while dopamine agonism exhibit anticonvulsant effects (Turski et al., 1988; Ogren and Pakh, 1993; Starr, 1996). Despite this complex and intricate puzzle investigating schizophrenic epilepsy comorbidity, the subject has gained significant attention, reflecting the debate on the possible common pathways (Kristensen and Sindrup, 1978; Wolf and Trimble, 1985; Diehl, 1989). There is a natural converging link between psychosis and epilepsy comorbidity, which is the impairment of limbic circuits, but the exact mechanism that promotes psychotic symptoms in epilepsy is still objected of intense discussion (Ma and Leung, 2002, 2004; Gutierrez-Galve et al., 2012; Nakahara et al., 2018; Scharfman et al., 2018). A group of researchers poses it is mainly related to the glutamatergic system, which could explain the change in hippocampal excitability and, consequently, the hyperactivity (Nissinen et al., 2000; Schobel et al., 2013; Bossong et al., 2019). The neurodegeneration of specific areas as the third layer of the medial entorhinal cortex is noted in patients and animal models of TLE that assessed psychotic-like behavior. In contrast, the GABAergic neurons remained more preserved than the glutamatergic ones (Kobayashi and Buckmaster, 2003). In this case, the loss of PV+ interneurons promotes, in the schizophrenia model, hyperactivity in the hippocampus and the hyper-responsivity of the dopaminergic system, and this converges to the findings that there is a substantial loss of parvalbumin protein without loss GABAergics neurons in the pilocarpine model (Benes, 2007; Lodge and Grace, 2007; Knopp et al., 2008; Heckers and Konradi, 2015).

On the other hand, it was proposed that in the animals treated with pilocarpine, the abnormality function of the hippocampus would be caused by alterations in the subicular inhibitory system as there is a reduction of glutamic acid decarboxylase (GAD) (Knopp et al., 2008) and loss of the PV+ interneurons in pyramidal cells (Guidotti et al., 2000). Highly convergent findings were reported in the TLE perforant electric kindling experimental model. The frequency of spontaneous seizures correlated with several psychotic-like behaviors and the PV+ interneurons density in the hippocampus was negatively correlated to the latency of Status Epilepticus and sensorimotor gating deficits (Wolf et al., 2016). Some of such reductions on GAD and parvalbumin findings were also seen in schizophrenia animal models and postmortem analysis of schizophrenic patients’ brains (Impagnatiello et al., 1998; Lodge et al., 2009; Cifelli and Grace, 2012).

GABAergic unbalance, mainly parvalbumin inhibitory activity, contributes to the sensorimotor gating deficits related to schizophrenia-like behaviors. Parvalbumin^–/–^ mice are less responsive to prepulse inhibition (PPI) than parvalbumin^+/+^ mice [Figure 1D(IV); Popelář et al., 2013]. However, other psychotic-like behavior parameters, such as locomotor activity, are similar in parvalbumin^–/–^ and parvalbumin^+/+^ mice (Wöhr et al., 2015). Parvalbumin^–/–^ mice are also more related to social behavior deficits, anxiety-like, and cognitive impairment, suggesting that these deficits could be related to autism spectrum symptoms. Parvalbumin knockout mice present less social interaction, reduced rearing activity in the center of the open field, and a deficit in reversal learning [Figure 1D(V); Wöhr et al., 2015]. This behavior in parvalbumin^–/–^ mice is related to neocortical hypertrophy in juveniles. Cognitive performance access by water maze is intact, as well as the sucrose consumption used to investigate anhedonia, indicating the complex relationship of PV+ interneurons in these knockout models (Wöhr et al., 2015).

Therefore, not only temporal areas seem to be related to this comorbidities’ behavior but also, there is an inhibitory reduction in the prefrontal cortex leading to an increased vulnerability for the development of psychosis (Beasley and Reynolds, 1997; Impagnatiello et al., 1998; Reynolds et al., 2002). Despite presenting a general situation pointing to GABAergic interneurons, the calcium-binding protein – calbindin, calretinin, and parvalbumin, represents 90% of these interneurons, and the parvalbumin by being expressed in two classes of neurons in local circuits, inhibiting, powerfully, the pyramidal neuron activity in the prefrontal cortex. Then, any loss in this circuit will create a relevant dysfunction; a fact noted in the schizophrenic brain (Benes et al., 1996; Tanaka, 2008).

Although it is still unclear how GABAergic and glutamatergic systems connect in this comorbidity puzzle, many discoveries compose and support the theory about the critical role of the parvalbumin inhibitory system and parvalbumin as the missing piece in psychosis behavior associated with epilepsy. Additionally, there are findings of interneuron dysfunction and myelination abnormalities of fast-spiking parvalbumin neurons (Stedehouder and Kushner, 2017). This scenario discusses the involvement of myelination of PV+ interneuron generating alterations in gamma oscillations frequency (30–100 Hz) relevant to working memory and attention (Senkowski and Gallinat, 2015). These findings are consistent in schizophrenic patients post-mortem samples evaluated by histopathology techniques (Gonzalez-Burgos et al., 2015).

## Intervention

For many years pharmacological interventions have been fundamental to treat and understand the etiology of epilepsy (Nirwan et al., 2018). However, new methods have helped unravel the inhibitory activity in seizures, especially PV+ interneurons (Forcelli, 2017; Magloire et al., 2019). In general, pharmacological intervention alters the neural network’s excitability and can be relatively safe and easy to deliver, but there are still high rates of pharmacoresistance (Kwan et al., 2011). In contrast, non-pharmacological intervention has become a promising approach to control seizures with more specificity. Nevertheless, there are still primary technique challenges to be clinically applied (Forcelli, 2017). The following sections will discuss these approaches in more detail (Figure 1E).

### Classical Antiepileptic Drugs Pharmacological Intervention

Considering that there is a loss of GABAergic inhibitory interneurons, pharmacologically blocking Na^+^ channels of those neurons may provide a basis for seizure aggravation. Na^+^ channel blockage by specific antiepileptic drugs (AEDs) as lamotrigine, carbamazepine in a scenario of an already compromised channel function in GABAergic interneurons, could increase network excitability (Guerrini et al., 1998; Hawkins et al., 2017). However, in a Dravet Na_*v*_ 1.1 knockout model, animals respond well to certain classes of AEDs as the ones that interfere in GABA_*A*_ receptors (Chiron and Dulac, 2011) which correlates to human therapeutic response [Figure 1E(I); Hawkins et al., 2017]. Other strategies could include enhancing GABAergic inhibitory neuron function through secondary mechanisms. For example, the selective activation of K_*v*_ 3.1 channels that underlie fast-spiking in specific GABAergic inhibitory neurons may help sustain parvalbumin activity and consequently reduce seizure susceptibility (Oyrer et al., 2018).

### Natural Compounds as Pharmacological Intervention

Some researchers hypothesize that preserving PV+ interneurons and their local circuit function by neuroprotective drugs could be a promising strategy to restore the functional network lost during the epileptogenic process.

Experimental studies (Khan et al., 2018) indicate that cannabidiol (CBD), a potential AED in some forms of refractory epilepsy, halts PV+ interneuron death in the hippocampi of KA-induced epileptic increase in parvalbumin-expressing cell densities and their dendritic length after CBD treatment. Also, CBD treatment produced a reduction of action potential threshold of PV+ cells *in vitro* Mg^2+^-free hippocampal brain slice model. The authors suggest that CBD restores normal network function by retrieving excitability and morphological impairments in epileptic models to pre-epilepsy control levels through multiple mechanisms to reinstate normal network function (Khan et al., 2018). Due to the myriad of mechanisms of actions in neurotransmitters, intracellular and anti-inflammatory pathways, it is difficult to establish the direct modulation of parvalbumin function in epilepsy and its comorbidities.

Another potential drug that exhibits significant neuroprotective effects against PV+ cell loss and anti-epileptic effects in the lithium-pilocarpine epilepsy model is the Parawixin2 molecule, whose main effect is to inhibit the uptake of GABA transporters (Godoy et al., 2017).

### Transcranial Magnetic Stimulation

The comprehension of parvalbumin mechanisms in epilepsies provides essential insights into new treatments and non-invasive approaches, such as transcranial magnetic stimulation (TMS) (Kimiskidis et al., 2014). TMS is an electromagnetic technique that can identify cortical inhibitory circuits by paired-pulse or achieve therapeutic effects by repetitive stimulation (Tang et al., 2017). It could be regarded as a new avenue previously paved by deep brain stimulation (DBS) discoveries. Many reports demonstrated that DBS in the hippocampus, amygdala, and cerebellum achieved significant success in controlling seizures in refractory patients (Klinger and Mittal, 2018).

Transcranial magnetic stimulation paired-pulses selectively can activate GABA inhibitory functioning, observed by short-interval of cortical inhibition and cortical silent period measures (Davies et al., 1990; Kujirai et al., 1993). Not surprisingly, patients with epilepsy exhibit specific alterations in cortical excitability assessed by TMS, as well as patients with psychiatric disorders, such as schizophrenia and depression (Bunse et al., 2013). Nonetheless, repetitive TMS can also increase cortical excitability and treat neuropsychiatric disorders (Tang et al., 2017).

In patients with partial epilepsy, repetitive TMS in the epileptogenic zone reduces seizures frequency and epileptiform discharges (Sun et al., 2012). Repetitive TMS for at least 4 weeks in psychiatric patients, reduced depressive symptoms (Bajbouj et al., 2006). However, despite the application of TMS, the mechanisms by which TMS modifies inhibitory circuits remain obscure. *In vitro*, repeated low-frequency TMS can induce long-term depression (LTD) (Tsumoto, 1992), while *in vivo* studies show that distinct patterns of TMS modulate different interneurons (Benali et al., 2011). These findings can support further non-pharmacological interventions in epilepsy focused on cortical inhibition, especially parvalbumin-related. Intermittent cortical theta-burst stimulation via TMS may affect the parvalbumin fast-spiking interneurons (Trippe et al., 2009; Benali et al., 2011; Volz et al., 2013) particularly this manipulation can interfere with cortical maturation, which is paralleled by intense growth of peri-neural nets and subsequent closure of the critical period (Hoppenrath et al., 2016). Also, there is a report on TMS patterned on endogenous thalamus-cortical bursting modulating the activation of PV+ interneurons (Huh et al., 2018).

Unlike deep stimulation, the risks of TMS are reduced because they do not involve an invasive surgical procedure and can be reversible/interrupted. Thus, this therapeutic focusing on PV+ interneurons activation could be considered for patients with a high level of refractoriness.

### Chemogenetics

Given the challenges and restrictions in pharmacological and surgical intervention, gene therapy has been considered the most promising treatment strategy to achieve unmet needs at the bedside and dissect the circuit’s function in physiology and behavior at the benchside (Maguire et al., 2014). In epilepsy research, strategies mostly rely on the expression of various proteins to prevent seizure initiation or propagation in targeted brain regions (Lieb et al., 2019). Some also have employed it to provoke seizures (Alexander et al., 2016). Chemogenetics involves altering cell pharmacological sensitivity by manipulating engineered receptors.

In the past, it has been explored chemogenetic modulation using allosteric modulation of the GABA_*A*_ receptor, as the allosteric sites (affinity for benzodiazepines drugs as zolpidem) have been genetically engineered to be rendered sensitive to pharmacological modulation in a restricted manner. Reversing this GABA_*A*_ receptor knock-in by generating zolpidem-insensitive mice and then genetically imposing zolpidem sensitivity on a selected cell type enables the manipulation of specific GABAergic circuits (Wulff et al., 2007). Thus GABAergic transmission could be enhanced or inhibited, and contextually modulated. One of the main limitations of utilizing this strategy involves the genetic background. It is also important to note that this approach does not allow for direct control of selected neurons, leading to vastly different responsiveness in various neuronal populations (Aldrin-Kirk and Björklund, 2019). Therefore this chemogenetic technique limits the potential applications for exclusively parvalbumin modulation.

By far, the most widely chemogenetic tool used is the DREADDs (Navabpour et al., 2020). The fundamental principle underlying DREADDs is that an engineered receptor has been mutated to render it insensitive to normal endogenous ligand (designer receptor) but sensitive to one or more exogenous compounds that otherwise have no effects on the tissue (designer drugs) for a profound understanding on the technique please visit further revision (Aldrin-Kirk and Björklund, 2019). Exogenous compounds can activate these receptors, and when expressed in neurons, can either inhibit or excite them (Armbruster et al., 2007). Glial cells have also been manipulated in fewer studies (Sweger et al., 2007).

The first receptors to be manipulated by the DREADD technique were G protein-coupled muscarinic receptors in cholinergic neurons (GPCR) (Scearce-Levie et al., 2001; Magnus et al., 2019). Those works have paved the way to chemogenetic intervention in many other receptors and cell types. To this present, molecularly circumscribed cell types (ranging from single synapses to the entire neuronal ensembles) can be manipulated as cell specificity can be achieved using *Cre*-inducible adeno-associated viruses expressing the designer receptor in combination with *Cre* recombinase expression (Smith et al., 2016; Ozawa and Arakawa, 2021). Also, transgene expression can be repressed upon administration of tetracycline or doxycycline, thus enabling it to halt or start cell activity (Das et al., 2016). Therefore parvalbumin cells can be either activated (hM3Dq/Ge-DREADDs) (Figure 1E(II)] or inhibited (hM4Di/Gi-DREADDs) by selective ligands within the spatial resolution and during some controlled period.

The most common drug design used in research is Clozapine-*N*-Oxide (CNO), an inert metabolite of the atypical antipsychotic drug clozapine together with olanzapine (Roth, 2016). In epilepsy, this tool can be used to understand the role groups of parvalbumin but normal and pathological mechanisms underlying the excitation-inhibition balance can be dissected. This knowledge could be further translated to drug design.

Some studies have assessed parvalbumin’s contribution to seizure generation and propagation through chemogenetics. The global silencing PV+ interneurons by intraperitoneal CNO injection in parvalbumin-Cre mice modified to express Gi-DREADDs induce behavioral arrest and generated absence-like seizures. In this study, the authors reported that CNO injection caused bursts of paroxysmal oscillatory discharges comprising spikes and wave-like discharges, with a frequency between 3 and 6 Hz. A behavioral arrest was associated with these bursts of oscillatory activity, with an increased mean frequency of such discharges dose-dependent. Only one animal out of nine had tonic-clonic seizures (Panthi and Leitch, 2019). The study observed the immobility behavior induced by DREADD manipulation, but also it promoted an increase in anxiety behavior. Authors additionally silenced PV+ interneurons via focal CNO injection into the somatosensory cortex or reticular thalamic nucleus, which induced similarly absence-like seizures associated with behavioral arrest (immobility state) and seizures presented with shorter latency compared to global silencing (Panthi and Leitch, 2019). Recently, the group showed DREADD-mediated activation of PV+ interneurons from the reticular thalamic nucleus and e somatosensory cortex provided anti-epileptic effects against PTZ-induced seizures. CNO activation of feedforward inhibition either prevented PTZ-induced or suppressed their severity (Panthi and Leitch, 2021).

It has been shown that focal DREADD silencing PV+ interneurons unilaterally in the ventral subiculum was sufficient to induce lasting seizures in the absence of cellular signs of neurodegeneration (Drexel et al., 2017). Specifically, parvalbumin/GABA prolonged silencing but not transient silencing (for ∼1–2 h) is sufficient to induce spontaneous recurrent seizures. The authors discuss the sustained designer drug inhibition results in reduced perisomatic feedforward inhibition *in vivo*, resulting in a decrease in seizure threshold with the development of cluster spike-wave discharges that spontaneous recurrent seizures could follow. Animals that only presented cluster spike-wave discharges were also more susceptible to seizures after a subthreshold PTZ injection (Drexel et al., 2011, 2017).

Another study that manipulated principal neurons in the forebrain by CNO administration to hM3Dq mice demonstrated this activation-induced enhanced gamma rhythm, seizures, and behavioral alterations. Interestingly, although it did not directly manipulate the interneuronal cell population, *in vivo* hippocampal recordings revealed that CNO produces an increased interneuron firing rate. They concluded that genetically activated excitatory pyramidal neurons promote synchronous interneuron firing (Alexander et al., 2009). Complementary to this finding, the inactivation of pyramidal neurons via the viral expression of a modified muscarinic receptor hM4Di produced an anti-ictiogenic effect (Wang et al., 2018) suppressed seizure induced by two different chemoconvulsants and in a chronic model of focal neocortical epilepsy (Kätzel et al., 2014).

The same study also investigated parvalbumin function to block seizure generation and saw that PV+ interneurons could be efficiently and specifically targeted with the excitatory DREADD receptor, hM3Dq. Specifically, the authors evaluated the seizure protective effect of pharmaco-genetic activation of hippocampal parvalbumin neurons (right ventral hippocampus) [Figure 1E(II)]. In the acute intrahippocampal kainic acid, it was observed that CNO 30 min before KA produced a dose-dependent increase in the latency to seizure, reducing both generalized tonic-clonic seizure and mortality. The same effects were shown in chronic models in hippocampal electrical fully kindled animals. When this DREADD intervention was tested in this chronic model, the activation of hippocampal PV+ interneurons reduced the seizure frequency and duration (Wang et al., 2018). Unfortunately, although the chemogenetic manipulation of parvalbumin activation could not significantly reduce observed deficits in learning and memory, it did not alter behavior and physical function in control groups. Thus, the study indicates that this pharmacogenetic activation of parvalbumin neurons may be relatively safe for normal physical function in control groups. This result suggests that epilepsy-associated cognitive and behavioral deficits might be mitigated only with a broader modulation of neuronal activity. This DREADD-mediated neuromodulation on seizure and behavioral phenotypes occurred 2 months after kainic acid administration. Research groups believe that earlier intervention could have achieved an even more robust effect (Wong and Escayg, 2019).

Another study also demonstrated in a combination of *in vitro* and *in vivo* studies in rodent models that chemogenetic enhancement of distinct populations of GABAergic interneurons parvalbumin activation reduced epileptiform discharges frequency in organotypic hippocampal slices model and increase in postsynaptic inhibitory input (which as observed for SST also) (Calin et al., 2018).

The authors discuss the prominent role of PV+ interneurons in postsynaptic inhibition due to their perisomatic targeting and extensive axonal trees. Therefore, individual PV+ interneurons are essential in mediating the effective inhibition of pyramidal neurons.

Interneuronal modulation by DREADD was evaluated in the 4-aminopyridine (4-AP) *in vivo* model. The 4-AP injection to one of the hippocampi resembles the clinical situation where focal seizures are frequently initiated in an area of limited abnormal brain tissue from where they spread and propagate through regular brain networks. Recruitment of hippocampal PV+ interneurons caused a reduction in the frequency in the more severe seizure behavior scores and reduced the occurrence of all convulsive behavior, thus probably blocking the generalization (Calin et al., 2018).

Reports show CNO pass the rodent blood-brain barrier (BBB), and a small amount of converted clozapine from systemic CNO delivery would presumably occupy CNS-expressed DREADDs *in vivo* (Gomez et al., 2017). There is limited data in primates (both human and nonhumans), but a few reports demonstrated similar findings in rodent research regarding designer drug delivery, activation, and long-lasting effect (Lieb et al., 2019).

For instance, some researchers have evaluated both CNO and clozapine effects in light of those new findings. In the study on parvalbumin activation in kainic acid and hippocampal kindled rats we described previously, the authors showed clozapine also significantly lowered seizure stage, shortened afterdischarges, and generalized tonic-clonic seizures. Interestingly, a seizure could not even be induced by the kindling stimulation in genetically modified drug-receptor mice that received clozapine pre-treatment (this was not observed in the CNO treated rats) (Wang et al., 2018). While this group argued this particular technological platform could be translated to humans, as three daily doses seem unlikely to be a potential problem (Wang et al., 2018), some other scientists face this manipulation with some concerns.

Designer drugs present some drawbacks that could be critical in translating those advances into the clinic. CNO is not a drug that has been approved for use in humans by the *Food and Drug Administration* (FDA) or *European Medicines Agency* (EMA) (Lieb et al., 2019). CNO metabolization into the psychoactive molecule clozapine may pose adverse side effects; therefore, the administration of CNO in DREADD mediated applications in both nonhuman primates and humans is an essential topic of investigation. Considering these regulatory issues, some studies discuss the subthreshold clozapine doses as an interesting approach as it is well studied and FDA approved (Gomez et al., 2017). However, clozapine receptor activation may be out of the question in clinical reports of epilepsy that show an increased incidence of seizures from both clozapine and olanzapine (Alper et al., 2007). In addition, there are some case reports of low-dose clozapine-induced seizures in non-epileptic patients (Bolu et al., 2017; Borah et al., 2019). To date, other hM3Dq agonist compounds that are very potent and quite selective (10,000-fold selectivity for hM3Dq over hM3) have been discovered, such as compound 21 and perlapine (Chen et al., 2015).

Even considering some potential drawbacks to the clinic, DREADD may be considered a promising intervention in epilepsy treatment (Hyder and Forcelli, 2020). This technique is not associated with potentially damaging instrumentation as intracerebral drug delivery, deep brain stimulation, or optogenetics, where devices must be physically present. Moreover, a relatively large area may be targeted, which is not limited by light absorption. Some studies have demonstrated that chemogenetics in nonhuman primates is feasible and explored the advantage of this technique in determining the location and density of receptor expression *in vivo* (when combined with functional magnetic resonance, PET scan) (Eldridge et al., 2016; Nagai et al., 2016). In addition to this advantage, post-mortem histochemical analysis in experimental models may corroborate and deepen the investigation. The slow onset and slower recovery (over optogenetics, for example) could be seen as a disadvantage, but for some types of behavioral experiments, especially at the clinic, this relatively long duration of action can be seen as an advantage.

### Optogenetics

*Optogenetics* is a methodology that enables the modulation of neurons or pathways through light-sensitive ion channel proteins or pumps expressed in the cells of interest through genetic manipulation (Deisseroth, 2011). Specific light lengths in these ion channels result in excitation (e.g., channelrhodopsin 2, ChR2) or neuronal inhibition (e.g., Archerhodopsin, Arch, or halorhodopsin, NpHR) with a high spatial and temporal resolution [Figure 1E(III)]. Other authors have addressed more detailed reviews on this subject (Deisseroth, 2015).

Optogenetic manipulations have also investigated the fundamental role of parvalbumin in recent decades, which allowed access to its causal relation (Jiang et al., 2017), with optimal temporal and spatial scale in both excitatory and inhibitory neurotransmissions (Boyden et al., 2005). The diversity of opsins from the publications of the first works with genetic engineering in producing transgenic animals has enabled the development of methods involving light stimulation. It has been allowed both to stimulate and inhibit the same neuron, to implement a rapid reversal with depolarization over a length of wave and hyperpolarization by a second color (Boyden, 2011), leading to the structuring of increasingly sophisticated activation silencing protocols of PV+ interneurons as induced in the work of Madisen et al. (2012) who used Cre-dependent mice for channelrhodopsin ChR2-tdTomato and ChR2-EYFP, halorhodopsin eNpHR3.0 and archaerhodopsin Arch-ER2.

Advances and improvements make it possible to mimic increasingly neurophysiological dysfunctions of various psychiatric diseases (Deisseroth, 2015), making such methods less invasive also without losing their specificity, as Matsubara and Yamashita (2021) indicated when reporting the development of new technologies that do not require the insertion of optical cannulas, just for the activation of transfected target cells stimulation with Near-Infrared-Mediated Optogenetics (NIR) (Bashkatov et al., 2005) which is an invisible light for animals and achieves surface of the cerebral cortex, but not in subcortical regions. However, opsins can be modulated in areas with large tissue volume employing X-Ray beams (Berry et al., 2015), contributing to the advantages of these new methods for behavioral experiments in rodents and clinical applications in neurological diseases.

In epilepsy basic research, optogenetic manipulation provided new insights into the role of parvalbumin in seizure onset and spread (Raimondo et al., 2019). Curiously, research with optogenetic manipulation showed contradictory results, while some studies have shown that parvalbumin activity decreased epileptiform activity, others have shown a pro-ictal effect [Figure 1E(III); Ledri et al., 2014; Yekhlef et al., 2015; Lee C. T. et al., 2016; Khoshkhoo et al., 2017; Shiri et al., 2017; Chang et al., 2018; González et al., 2018]. PV+ interneuron optogenetic stimulation is anti-ictogenic, however, its stimulation can paradoxically trigger seizures in epileptic animals, supporting the notion that the enhanced inhibitory signaling can also initiate ictogenesis (Lévesque et al., 2019). However, parvalbumin can play distinct roles in seizures, depending on its spatial position or seizure progression. In the temporal cortex slices, modulation of parvalbumin activity in epileptic focus is insufficient to prevent epileptogenesis. Nonetheless, parvalbumin optogenetic modulation in distal sites can block ictal spread and reduce its duration (Sessolo et al., 2015). Similarly, optical stimulation of thalamic parvalbumin decreases the duration of generalized seizures in the PTZ model (Clemente-Perez et al., 2017). Specifically, the CA1 hippocampus (Miri et al., 2018) shows a distinct parvalbumin activity pattern during preictal and ictal periods. There is a preictal increased PV+ interneuron firing, followed by an ictal decreased PV+ interneuron firing. In addition (Magloire et al., 2019) demonstrated that parvalbumin optic stimulation could switch from an anti-ictal to pro-ictal after seizure initiation. The author suggests that after seizure onset, GABAergic signal switches to an excitatory signal through disarrangement of chloride extruder. Besides these valuable recent discoveries, the role of parvalbumin in epilepsy has not yet been unraveled. Still, optogenetics remains a valuable tool for understanding epilepsy etiology and a future clinical approach to suppress seizures.

Much akin to current deep brain stimulation approaches, optogenetics may be theoretically deployed in a self-contained manner with implanted fibers and stimulators requiring an initially invasive surgery followed by no further need for tethering or drug treatment (Forcelli, 2017).

### Clustered Regularly Interspaced Short Palindromic Repeats/Cas9

Altering the genetic landscape related to epilepsies is a highly challenging task. Not only does it demand a high degree of caution, but also many epilepsies involve polygenic mutations and genes that regulate fundamental processes in the brain. *Programmable DNA-binding agents* (PDBAs) can be targeted to any locus in the genome. Particularly, *transcription activator-like effector* (TALE) and *Clustered Regularly Interspaced Short Palindromic Repeats* (CRISPR)/CRISPR associated (Cas) systems can be targeted at nucleotide resolution (Lee H. B. et al., 2016).

With successive innovations, the CRISPR/Cas9 system has become widely adopted for genome engineering and is increasingly regarded as a clinical intervention for specific genome editing (Lee H. B. et al., 2016). The CRISPR technique is highly relevant to the field of genetic epilepsy as either dominant heterozygous pathogenic missense variation or small insertion/deletion could be targeted for correction using this technique (Goldberg, 2020).

One of the many advances of this technique is that gene targeting requires a custom single guide RNA (sgRNA) production that can be easily prepared with one cloning process. The technique is time- labor-, and cost-effective. Mediated by a small guide RNA (sgRNA or gRNA), Cas9 can specifically introduce double-strand DNA breaks at pre-selected genomic loci, which (i) contain a target sequence (protospacer) complementary to the typically 20 nucleotides guide sequence of the gRNA, and (ii) are followed by a protospacer sequence adjacent motif. By the co-delivery of Cas9 and multiple gRNAs, the CRISPR/Cas9 system enables the simultaneous modification of several loci/genes (Cong et al., 2013; Lin et al., 2016).

Therefore, the simplicity of sgRNA production has made it possible to generate gene libraries, gain and loss of valuable function for genetic screens and genetic therapy, which can be applied for either human, mouse, and primates studies (Cong et al., 2013). This demonstrates that features of given genetic epilepsy can be treated or even prevented (Goldberg, 2020).

Colasante et al. (2020) delivered an AAV9-based *Scn1a*.dCas9 system targeted to GABAergic interneurons via intracerebroventricular injection into neonatal *Scn1a*^+/–^ mouse pups. Electrophysiological recordings demonstrated that deficits in action potential generation in parvalbumin fast-spiking GABAergic interneurons known to be dysfunctional in *Scn1a*^+/–^ mice were rescued by the *Scn1a*.dCas9 system. This report shows new horizons on the promising intervention by the CRISPR/Cas9 system in parvalbumin cells, which can promote a significant advance in epilepsy therapy.

### Cell Therapy

Given the abnormalities of GABAergic inhibitory interneurons in epileptic brain tissues, a strategy with considerable promise is to restore normal circuit function by transplanting GABAergic interneurons/progenitors to the seizure focus (Zhu et al., 2018). Interneuron cell transplantation is a powerful approach to halting seizures and rescuing accompanying deficits in severe epilepsy in animal models [Hunt et al., 2013; Figure 1E(IV)].

During early brain development, cortical GABAergic interneurons primarily arise from Medial Ganglionic Eminence (MGE) and Caudal Ganglionic Eminence (CGE) and then migrate tangentially over long distances to their final destinations, where they form local inhibitory synaptic connections with excitatory pyramidal neurons and other GABAergic interneurons (Tyson and Anderson, 2014).

Parvalbumin-expressing cells derive from MGE *Nkx2.1*-expressing progenitors, and a series of other transcription factors regulate the migration, differentiation, and electrophysiological properties (Zhao et al., 2008; Okaty et al., 2009). Studies have mapped in experimental models if transplantation of MGE cells would recapitulate the adequate differentiation and fate process. In mouse brains, the transplantations of MGE progenitor’s embryonic day into the postnatal neocortex were tested. MGE-derived neurons are preferentially and densely distributed in neocortical and, as expected, differentiated into PV+ and somatostatin-positive interneurons within Layers 2/3, 5, and 6. Those findings showed an anatomical integration of MGE-derived interneurons following transplantation (Sebe et al., 2014). Those cells not only do exhibit the morphological and neurochemical features of interneurons, but they also fire like mature interneurons (Sebe and Baraban, 2011). Some protocols were optimized to improve grafted cells survival and differentiation rates (Franchi et al., 2016; Wang et al., 2016) and showed a significant increase in GABA-mediated synaptic inhibition (Alvarez-Dolado et al., 2006). Derivation of grafted cells into interneurons can also be achieved using human pluripotent stem cells (hPSCs). Reports also demonstrate the potential for clinical translation (examining patient individual characteristics) and foster quality-controlled human cell research that can enhance inhibitory drive and restore dysfunctional host circuitry. In humans, it is usually employed the differentiation of MGE progenitor cells from pluripotent stem cells (PSCs), embryonic stem cells (ESCs), and induced pluripotent stem cells (iPSCs) (Rao et al., 2017), which are similar to MGE-grafted cells. They demonstrate functional synaptic integration in the host region and represent a promising clinical intervention (Cunningham et al., 2014). For additional information, we suggest some other great works (Hunt and Baraban, 2015; Shetty and Upadhya, 2016; Rao et al., 2017).

Interestingly, the new therapy horizon considers combining MGE cell transplantation with one of the different techniques listed in this review. For instance, modified MGE has been associated with selectively activated by DREADD or optogenetics in a system that could represent a self-regulating closed-loop where grafted interneurons could be specifically activated during periods of heightened hyperexcitability. Those engrafted cells associated with other anticonvulsant mechanisms would render interneurons as an ultimate tool in seizure (Zhu et al., 2018).

Interneuronal cells derived from MGE were transplanted in the transgenic mouse model of channelopathy associated with epilepsy that presents loss-of-function of a Shaker-like potassium channel *Kv1.1*/*Kcna1*. Mice grafted with MGE cells bilaterally in the cortex (P32–P39) exhibited significantly reduced electrographic seizure activity and duration compared with untreated or vehicle-injected mutants. Immunohistochemistry analysis revealed MGE cells differentiate mainly in somatostatin and PV+ interneurons (44 and 29%, respectively), and the antiepileptic properties were attributed to the enhancement of GABA-mediated inhibition onto host pyramidal neurons, based on electrophysiology *in vitro* and immunohistochemical techniques (Baraban et al., 2009).

Newly generated MGE inhibitory neurons (of which 30% are PV+) were distributed throughout the adult hippocampus of adult epileptic mice in the pilocarpine TLE model. The group that received grafted interneurons showed a marked reduction in the electrographic seizures and restored behavioral deficits in spatial learning, hyperactivity, and the aggressive response to handling. In the host brain, GABAergic progenitors migrated up to 1,500 μm from the injection site, and in addition to expressing genes and proteins characteristic of interneurons, they also differentiated into functional inhibitory neurons and received excitatory synaptic input. In contrast with the hippocampus, from cell grafts into the basolateral amygdala, about only 10% differentiated into interneurons (the majority was somatostatin-positive). Although the group that received grafted cells rescued the hyperactivity deficit, the treatment did not affect seizure frequency (Hunt et al., 2013).

Stem cells from the anterior subventricular zone (SVZ) of postnatal F344 rat pups expressing the human placental alkaline phosphatase were grafted into the hippocampus of young adult rats at 5 days after excitotoxic injury (event-related in many of the so-called “acquired” epilepsies). Analyses through the forced swim, water maze, and novel object recognition tests revealed SVZ stem cells grafting reversed the injury-induced cognitive deficit and depressive-like state. Graft-derived cells exhibited excellent survival and pervasive migration, and they mainly differentiated into astrocytes and GABAergic interneurons, including neurons and oligodendrocytes. Interneuronal types were mainly characterized by calbindin+ and PV+ reductions and abnormalities in neurogenesis by both maintaining a normal level of stem cell activity in the subgranular zone. Grafted cells expressed significant amounts of neurotrophic factors, which were believed to contribute to stimulating neurogenesis in both the local injured area and subventricular zone, probably contributing to the increased reelin secretion, an extracellular matrix protein provided by interneurons that control newly born dentate granule cell migration (Hattiangady and Shetty, 2012).

Similar findings were observed in the pilocarpine chronic TLE. The research group that has previously provided a very descriptive protocol using this model (Hattiangady and Shetty, 2011) reported the effects of hiPSC-derived MGE-like interneuron precursors grafting into rat hippocampus after status epilepticus (SE). They demonstrated that the treatment could significantly reduce spontaneous seizures in the chronic phase through antiepileptogenic and antiepileptic effects by reducing seizure frequency and duration from the first week after transplantation. By examining anxiety (elevated plus maze), depressive-like (forced swim test) behaviors, and short-term memory impairment in epileptic rats, they observed that the SE grafted group restored the cognitive and mood function. It was demonstrated that grafted cells differentiated mature neurons, and from those, some were interneurons, mostly PV+ (27%) and neuropeptide Y-positive (11%).

Interestingly, graft-derived neuropeptide Y-positive and somatostatin-positive interneurons were primarily seen in smaller clusters, suggesting specific clones probably derived them. Grafted groups exhibited a smaller reduction of normal neurogenesis induced by SE, also displaying fewer aberrant neurogenesis. Animals submitted to cell therapy presented a smaller reduction in reelin interneurons, preserving more PV+, neuropeptide Y+, and somatostatin+ interneurons 5 months after SE. To test whether all the therapeutic effects were related to the activity of grafted cells, a different group received the transplanted cells that were genetically modified for inhibitory modulation by DREADD protocol. It has compared a period of inhibition of grafted cells by CNO administration with a previous control period. Researchers showed the seizure suppression was abolished, suggesting a direct antiepileptic impact of graft-derived interneurons through increased inhibitory neurotransmission (Upadhya et al., 2019).

Comparing this study and previously published by Hunt and colleagues, as both used pilocarpine TLE models, it would be interesting to discuss the differences, implications, and the potential advances of using inhibitory human pluripotent stem cells.

Shetty’s research group recently expanded the translational use of grafted cell therapy using a non-invasive intranasally administered Human MSC-Derived Extracellular Vesicles protocol. They developed a protocol for efficiently delivering extracellular vesicles, nanosized membranous particles released by neural stem cells, and mesenchymal stem cells from the bone marrow (hMSCs). Intranasally Administered hMSCs incorporation by neurons were comparable between Naïve and SE Rats in most forebrain regions, but interestingly, SE-injured lesions incorporated more cells (Somatosensory Cortex, CA1, and entorhinal cortex). Extracellular Vesicles markers were mostly found inside neurons and microglia but not within astrocytes, and it was not reported if hMSCs differentiated into interneuronal subtypes (Kodali et al., 2019). A previous paper from the laboratory has demonstrated intranasal of extracellular vesicles hMSCs administration 24 h after SE reduced glutamatergic and GABAergic neuronal loss, significantly reduced inflammation in the hippocampus, and showed long-term preservation of normal hippocampal neurogenesis and cognitive and memory function (Long et al., 2017).

## Future Directions

Considering the significant role in regulating many physiological processes, such as intracellular signaling and synaptic transmission, changes in parvalbumin are deeply related to epilepsy. This review discussed some preclinical evidence from various animal models in epilepsy that demonstrate the translational value of interventions aiming at those interneurons.

We have explored different forms of manipulations, from current antiepileptic and neuroprotective drugs acting on PV+ interneurons to cutting-edge manipulations such as DREADD and optogenetics, cell therapy, or other groundbreaking advances in neurosciences. Those techniques can be viewed as therapeutic approaches and innovations that enable us to move up to a higher understanding of the pathophysiology of the disease. The current state of DREADD has significantly advanced toward synthetic improved chemical ligands such as JHU37152 and JHU37160, more potent and selective that can be associated with high-affinity DREADD PET radiolabeled ligands and be mapped through non-invasive visualization, additionally to DREADD-assisted metabolic mapping (Bonaventura et al., 2019). Those are currently being tested in primates and constitute a promising translational approach. Other advances are noted as a clinical intervention but also as better optimized tools in basic neuroscience research. Recently it has been described as new platform for the DREADD technique, the designed K opioid receptor (KOR) which can be selectively inhibited by the inert drug salvinorin B (SALB). This is quite interesting because adding a different platform enables the co-expression of the KORD and the Gq-coupled M3-DREADD within the same neuronal population, thus allowing a bidirectional remote control (Vardy et al., 2015).

Similarly, there are advances in optogenetics that enable the dual control of excitation and inhibition. BiPOLES is a reliable dual-color neuronal spiking and silencing optogenetic tool that was already demonstrated in worms, flies, mice, and ferrets (Vierock et al., 2021). The practical implementation of optogenetic in the clinic is associated with a combination of challenges, some of which are novel and some of which have precedent in the development of other clinical treatments. A key component of safety in optogenetics in humans is not just how neurons respond to viral injections, immunological reaction, or the implantation of a device, but how the cells react upon exposure to light, its variation to intensity and heat. For example, precedent for aspects of optogenetic therapies can be found in gene therapy, and chronic brain implants used for closed-loop Deep Brain Stimulation (DBS) systems (Shen et al., 2020). Some clinical trials have already been initiated focusing on restoring vision function involving AAV genes delivery and require access to specially designed goggles to deliver light to the treated eye (Harris and Gilbert, 2021). Solutions to this problem have mainly consisted of modifying the design of LEDs and lasers, as well as optical fibers, but these are invasive, require electrical circuits that may fail, and can heat the tissue if not appropriately calibrated (Owen et al., 2019). A new step-function opsin with ultra-high light sensitivity (SOUL) has been recently developed and consists of a minimally invasive tool for manipulating neuronal activity from outside the dura. The technology was tested in knock-in mice and primates and adds an important step toward implementation in humans (Gong et al., 2020).

As previously discussed, many pharmacological treatments present a non-specific mechanism that makes it difficult to pinpoint the role and changes in parvalbumin related to the pathology and the antiepileptic effect. All drugs indicated in this review have been shown to generally act on GABAergic neurotransmission, which is the most studied and evaluated in different treatment protocols. Those are valuable and indispensable interventions to the clinic and understand inhibitory transmission in the pathophysiology, but non-pharmacological approaches may represent a new horizon of neuroscience research, including epileptology.

Embryonic mesenchymal cells differentiate mainly in somatostatin and PV+ interneurons, and the antiepileptic properties were attributed to the enhancement of GABA-mediated inhibition onto host pyramidal neurons based on the many reports described here. In 2020, Cronutt, a 7-year-old male sea lion with intractable seizures due to algae intoxication, successfully received a precisely targeted injection of MGE-cell from pig source in the hippocampus. Since then, Cronutt has been seizure-free and this report has paved a new avenue for cell therapy in large mammals (Weiler, 2020).

Reduced numbers of parvalbumin cells, altered morphology, immature electrophysiological properties with lower firing rates, and power of gamma oscillations play a role in Epilepsy and its comorbidities. Strategies of enhancing GABAergic inhibitory neuron function either through secondary mechanisms or directly by modulating parvalbumin function may affect its activity and consequently reduce seizure susceptibility and behavioral disruption. Similarly, TMS pulses interfere with GABAergic functioning and possibly the parvalbumin fast-spiking interneurons changing synchronization and cortical excitation that may present abnormally in patients with epilepsies and/or psychiatric disorders.

Finally, since parvalbumin is involved in many higher cognitive processes, its altered function is related to other psychopathologies and psychiatric comorbidities in Epilepsy. There are some challenges in addressing behavior and comorbidities in experimental epilepsy studies. Many of the described findings showed differences in some behavioral tests associated with many essential processes and not to a specific phenotype involved in anxiety, depressive, and psychotic-like behavior. Untangling differences and understanding the mechanism and changes in brain structures may contribute to a neurobiology perspective in epilepsy comorbidities. Parvalbumin functioning requires such an approach due to its intrinsic characteristics of regulating information processing through different brain regions.

Considering this, chemogenetic and optogenetics approaches could provide essential insights into parvalbumin’s role in Epilepsy and psychiatric comorbidities. In rodents, stimulation of PV+ interneurons shows a complex engram of circuits related to psychiatric-like behaviors. Depressive-like behaviors are mainly related to disruption of prefrontal parvalbumin activity, but the hippocampus contributes at least in part to this process. In contrast, the hippocampus plays a fundamental role in anxiogenic and psychotic-like behaviors, with the amygdala and prefrontal cortex also associated with anxiety and psychotic-like deficits, respectively. Still, parvalbumin activity in several not-limbic structures could influence these effects and be investigated in the future. Nevertheless, it is possible to speculate that the propagation of seizures in Epilepsy may affect the functioning of PV+ interneurons in different circuits, sharing similar circuits that are involved in psychiatric symptoms exclusively.

## Author Contributions

LG, TP, MR, and JLL conceived and wrote the manuscript. MR designed the figure. JPL contributed with a critical and historical revision, and with funding acquisition. All authors approved the manuscript for publication.

## Conflict of Interest

The authors declare that the research was conducted in the absence of any commercial or financial relationships that could be construed as a potential conflict of interest.

## Publisher’s Note

All claims expressed in this article are solely those of the authors and do not necessarily represent those of their affiliated organizations, or those of the publisher, the editors and the reviewers. Any product that may be evaluated in this article, or claim that may be made by its manufacturer, is not guaranteed or endorsed by the publisher.
