# Supplementary material for: Parvalbumin Role in Epilepsy and Psychiatric Comorbidities: From Mechanism to Intervention
Source: Front Integr Neurosci. 2022 Feb 17;16:765324. doi: 10.3389/fnint.2022.765324 (PMC8891758; doi:10.3389/fnint.2022.765324)
Supplement: Supplementary file 1 [file Data_Sheet_1.docx]

Supplementary Material

We used the following combined descriptors for prospecting the articles in the PubMed (https://www.ncbi.nlm.nih.gov/) database from the 1980s to 2021 to produce this review article: epilepsy(ies) or seizure(s) with parvalbumin, parvalbuminergic, or parvalbumin with terms related to the respective sections.

In the experimental section were used the terms "*chemical model*", "*electric model*", "*animal model of epilepsy*", "*animal model of seizure*", "*convulsant*", "*receptor or protein mutation*", "*Gain and loss of function OR knockout*", "*genetic strain* OR *seizure prone*", "*Development Mutation*". Comorbidities section included "*psychiatric comorbidity(ies)*", "*schizophrenia OR psychosis*", "*depression* OR depressive" and "*anxiety*") terms.

The intervention section included terms related to the respective subsection themes. In pharmacological search used "*pharmacological*", "*antiepileptic drugs*", "*anticonvulsant*" including another specific search of GABaergic drugs. In non-pharmacological subsection "*Receptor Activated Solely by a Synthetic Ligand OR RASSL*" "*Designer Receptor Exclusively Activated by Designer Drugs OR DREADD*", "*Transcranial magnetic stimulation or TMS*", "*Optogenetic*", "*Programmable DNA-binding agents OR PDBAs*" AND "*transcription activator-like effector OR TALE*" AND "*Clustered Regularly Interspaced Short Palindromic Repeats OR CRISPR OR CRISPR Cas9*", "*MGE-derived neurons*", "*grafted cell, human pluripotent stem cells OR hPSCs*", "*induced pluripotent stem cells OR iPSC*s", "*progenitor cells from pluripotent stem cells OR PSCs*" "*embryonic stem cells OR ESCs*."

Searches were narrowed to Title/Abstract, and other independent and non-structured methods of search and revision were implemented in some subsections.
